# Associations of Comorbid Atopic Dermatitis, Food Allergy, and Respiratory Allergic Diseases with Somatic Problems in Children: A Birth Cohort Study

**DOI:** 10.3390/children13081017

**Published:** 2026-07-30

**Authors:** Sungsu Jung, Jisun Yoon, Kyungmo Hong, Hea Young Oh, Eom Ji Choi, Min Jee Park, Song-I Yang, Eun Lee, Hyo-Bin Kim, So-Yeon Lee, Soo-Jong Hong, on behalf of the PSKC Study Group

**Affiliations:** 1 Department of Pediatrics, Pusan National University Yangsan Hospital, Pusan National University School of Medicine, Yangsan 50612, Republic of Korea; jung5984@pusan.ac.kr; 2 Department of Pediatrics, Asan Medical Center, Seoul 05505, Republic of Korea; jsyoon@amc.seoul.kr; 3 Psychotherapy, Graduate School of Health and Welfare, Baekseok University, Cheonan 31065, Republic of Korea; kyungmo@bu.ac.kr; 4 Department of Pediatrics, Humidifier Disinfectant Health Center, National Medical Center, Seoul 04564, Republic of Korea; helloaz@nmc.or.kr; 5 Department of Pediatrics, CHA Gangnam Medical Center, CHA University School of Medicine, Seoul 06135, Republic of Korea; allergy2024@chamc.co.kr; 6 Department of Pediatrics, Hallym University Sacred Heart Hospital, Hallym University College of Medicine, Anyang 14068, Republic of Korea; 230001@hallym.or.kr (M.J.P.); ysi222@hallym.or.kr (S.-I.Y.); 7 Department of Pediatrics, Chonnam National University Hospital, Chonnam National University Medical School, Gwangju 61469, Republic of Korea; unelee@jnu.ac.kr; 8 Department of Pediatrics, Inha University Hospital, Inha University School of Medicine, Incheon 22332, Republic of Korea; hbkim1126@inha.ac.kr; 9 PHI Digital Healthcare, Seoul 03722, Republic of Korea; 10 Institute for Innovation in Digital Healthcare, Yonsei University, Seoul 03722, Republic of Korea

**Keywords:** dermatitis, atopic, food hypersensitivity, mental disorders, somatic symptom disorder, behavior rating scales, cohort

## Abstract

**Highlights:**

**What are the main findings?**

- In a population-based birth cohort of 1577 seven-year-old children, atopic dermatitis (AD) and food allergy (FA) were each associated with DSM-oriented somatic problems, and children with comorbid AD and FA showed markedly higher odds than those with either condition alone.
- Allergic rhinitis statistically accounted for part of the association between AD and somatic problems, and the direct association of AD persisted after accounting for respiratory allergies.

**What are the implications of the main findings?**

- Allergic multimorbidity—particularly coexisting AD and FA—may warrant integrated clinical attention that includes screening for somatic and psychological problems, alongside management of the skin and nasal symptoms.
- Because exposures and outcomes were assessed concurrently, these associations are exploratory and hypothesis-generating rather than causal, and warrant confirmation in prospective studies.

**Abstract:**

Background/Objectives: Atopic dermatitis (AD) and food allergy (FA) frequently coexist in the concept of atopic march; however, their combined associations with psychological problems remain poorly understood. This study aimed to investigate the associations of AD and FA with psychological outcomes at age 7 and evaluate whether respiratory allergies account for part of these associations. Methods: We enrolled 1577 children aged 7 years from the Panel Study of Korean Children. The Korean version of the Child Behavior Checklist was used to assess psychological and behavioral problems. Multivariable regression and parallel mediation analyses were used to evaluate independent and interactive associations. Results: AD and FA were associated with psychological and behavioral problems, most consistently with somatic problems on the Diagnostic and Statistical Manual of Mental Disorders (DSM)-oriented scale. These associations were more pronounced in children with comorbid AD and FA than in those with single allergies. Among the 9 children with concurrent AD and FA, an interaction between the two conditions on somatic problems was observed (*P* for interaction = 0.033). The comorbid group showed higher eosinophil counts and total IgE levels, but no differences in SCORAD indices or C-reactive protein levels. Mediation analysis identified a direct association of AD with somatic problems (*B* = 1.062; 95% CI, 0.601–1.562), with a portion accounted for by allergic rhinitis (AR) (*B* = 0.656; 95% CI, 0.306–1.241). Conclusions: Comorbid AD and FA were associated with higher odds of somatic problems than either condition alone in 7-year-old children, with a portion of the AD association accounted for by AR. Because exposures and outcomes were assessed concurrently, these associations are exploratory and hypothesis-generating rather than causal. Clinical attention may be warranted for children with both AD and FA.

## 1. Introduction

The prevalence of allergic diseases, including atopic dermatitis (AD) and food allergy (FA), has increased markedly among children worldwide in recent decades [1,2]. Beyond their physical symptoms, these conditions substantially impair the quality of life of affected children and their families, often causing sleep disturbances, financial burden, and increasing family stress [3,4,5].

Numerous studies have demonstrated significant associations between childhood allergic diseases and mental health disorders, including depression, anxiety, and attention-deficit/hyperactivity disorder (ADHD) [6,7,8]. Emerging evidence on the gut–lung–brain axis further suggests that systemic allergic inflammation may be linked to neurodevelopmental, psychological, and behavioral difficulties in children [9,10].

Although AD and FA have been associated with increased risks of anxiety and ADHD [11,12,13,14], these conditions frequently coexist as manifestations of the atopic march [2,15]. Despite their frequent co-occurrence, the combined effects of AD and FA on psychological health remain poorly understood. It is unclear whether their coexistence produces a psychological burden greater than the sum of their individual effects, representing a key gap in understanding allergic multimorbidity.

The progression of the atopic march to asthma and allergic rhinitis (AR) further complicates this relationship [16,17]. Whether these respiratory allergic comorbidities act as mediators, independent contributors, or interacting factors in psychological distress remains unclear. Clarifying these pathways is essential for developing comprehensive management strategies that address both physical symptoms and mental well-being.

Therefore, using a general population-based birth cohort, we aimed to investigate the independent and combined associations of AD and FA with psychological and behavioral outcomes in 7-year-old children using the Child Behavior Checklist (CBCL). Because associations were most consistent for somatic problems, this domain is the focus of the present report. We also examined the potential contribution of respiratory allergic comorbidities, specifically asthma and AR, to better understand the pathways underlying these associations.

## 2. Methods

### 2.1. Study Participants

The Panel Study of Korean Children (PSKC) is a nationwide population-based birth cohort previously established to provide longitudinal data on childhood development [18]. The PSKC birth cohort recruited 2078 mother–baby dyads using two-step stratified random sampling across Korea in 2008 and collects developmental information and longitudinal data on allergic diseases [19,20]. The CBCL is used to measure cognitive and non-cognitive abilities. In total, 1577 participants were followed up in 2015 and completed the allergy questionnaire and CBCL, and constituted the analytic sample (Figure S1). The prevalence of each allergic disease is presented in Table S3, and the sex distribution of the analytic sample is presented in Tables S1 and S2. Among these participants, allergic and systemic inflammatory biomarkers—including C-reactive protein (CRP), blood eosinophil counts, and total IgE—together with the SCORAD index, were measured in 633 children who attended one of the 16 participating regional hospitals, whereas the remaining 944 children did not attend. Supplementary Table S1 presents the characteristics of the participants who visited the regional study hospitals compared to the PSKC participants who declined these visits.

The Institutional Review Board of Asan Medical Center reviewed and approved the study protocol (No. 2015-0907). Written informed consent was obtained from all parents and guardians following a detailed explanation of the study.

### 2.2. Definition of Allergic Disease Phenotypes

To evaluate allergic diseases, we employed the ISAAC-based questionnaire, which is widely recognized and validated in large-scale epidemiological studies [21]. Diagnosis-ever for AD, FA, asthma, and AR was defined as a physician-confirmed diagnosis by age 7, based on parental reports. Current status for each condition was further determined by symptoms or management within the preceding 12 months: current AD required at least one eczema episode; current FA necessitated an elimination diet lasting more than 6 months; and current asthma or AR was defined by at least one wheezing episode or rhinitis symptoms, respectively.

### 2.3. Psychological and Behavioral Problems

The CBCL is a widely utilized parent-reported instrument designed to assess emotional and behavioral symptoms in children [22]. The age 6–18 version of the checklist was employed to evaluate psychological and behavioral health through syndrome scales (categorized into internalizing and externalizing problems), Diagnostic and Statistical Manual of Mental Disorders (DSM)-oriented domains, and specialized measures for social, cognitive, and trauma-related symptoms. The reliability and validity of the CBCL have been extensively documented in the Achenbach Manual [22], and the Korean version (K-CBCL) has demonstrated robust psychometric properties [23]. Details regarding the assessment of psychological and behavioral problems are provided in the Supplementary Online Materials.

### 2.4. SCORing Atopic Dermatitis (SCORAD) Index Assessment

Pediatric allergy specialists assessed the SCORAD index in participants with AD [24]. Moderate-to-severe AD was defined as a SCORAD index of ≥25. The participant flow is shown in Figure S1. Among the 633 children who attended a regional hospital at 7 years of age, 74 had current AD, and 37 of these had an available SCORAD index (Table S2). Because the SCORAD index was available for only 37 children, analyses involving disease severity were regarded as exploratory.

Baseline characteristics were compared between participants who underwent SCORAD assessment and those who did not (Supplementary Table S2). No significant differences in baseline characteristics were observed between the two groups.

### 2.5. Potential Mediators

We examined the mediating and moderating roles of respiratory allergic comorbidities, specifically asthma and AR, in the association of AD and FA with psychological and behavioral outcomes, respectively. In this final multivariate model, comorbid respiratory allergies (asthma and AR) were adjusted for as covariates to isolate the primary association between AD and FA by controlling for these potential confounders.

### 2.6. Statistical Analysis

Statistical analyses were performed using SPSS (version 23; IBM Corp., Armonk, NY, USA) and Stata version 14.2 (StataCorp LLC, College Station, TX, USA). Chi-square or Fisher’s exact tests were used to compare the characteristics between participants who visited a regional hospital and those who did not, as well as between participants categorized by the presence of a SCORAD index or moderate-to-severe AD. Logistic regression analysis was used to investigate the association between allergic disease phenotypes and psychological and behavioral problems. Adjusted odds ratios (aORs) and 95% confidence intervals (CIs) were calculated after controlling for potential confounders, including sex, maternal education level, parental allergy history, second-hand smoke exposure, residential area, and socioeconomic status. For sex-stratified subgroup analyses, the same set of confounders was adjusted, excluding sex.

Student’s *t*-tests were used to compare the CBCL T-scores between children with and without allergic diseases. Mann–Whitney U tests were used to compare SCORAD indices between the groups based on the presence of FA, current FA, and somatic problems. Furthermore, parallel mediation analyses were conducted to assess whether the associations of AD and FA with somatic problems were mediated by asthma and AR. These analyses used logistic regression-based models, and the significance of the indirect effects was confirmed via bootstrapping (1000 resamples) in Python v3.10.

## 3. Results

### 3.1. Prevalence of Allergic Diseases in PSKC

Among the children in the cohort, the prevalence rates of AD and FA were 20.0% and 5.6%, respectively. When categorized according to phenotype, the prevalence rates of AD-ever without FA, FA-ever without AD, comorbid AD and FA were 17.6%, 3.2%, and 2.4%, respectively. Regarding respiratory allergies, the prevalence of diagnosis was 5.8% for asthma and 45.2% for AR. The prevalence of current allergic conditions was lower than that of lifetime diagnoses as follows: current AD (8.6%), current FA (2.0%), current AD only (8.0%), current FA only (1.4%), and comorbid current AD and FA (0.6%). Additionally, the prevalence rates of current asthma and AR were 1.5% and 32.2%, respectively (Supplementary Table S3). Attendees at a regional hospital (*n* = 633) and non-attendees (*n* = 944) did not differ in general characteristics except for a higher rate of prior bronchiolitis among attendees (24.4% vs. 18.3%, *p* = 0.004; Table S1).

### 3.2. Allergic Diseases and Psychological/Behavioral Problems

Diagnosis-ever of AD was significantly associated with somatic complaints on the behavior problem scale (aOR, 2.35; 95% CI, 1.33–4.13), as well as somatic problems (aOR, 2.94; 95% CI, 1.78–4.87) and ADHD (aOR, 1.95; 95% CI, 1.08–3.52) on the DSM-oriented scales. Similarly, FA diagnosis was associated with DSM-oriented somatic problems (aOR, 2.98; 95% CI, 1.45–6.14). Regarding current status, both current AD (aOR, 2.62; 95% CI, 1.39–4.93) and FA (aOR, 5.40; 95% CI, 2.09–13.97) were significantly associated with DSM-oriented somatic problems (Table 1).

Regarding CBCL T-scores, children with a diagnosis-ever of AD exhibited significantly higher scores than those without for internalizing problems (51.0 ± 9.3 vs. 49.3 ± 8.6; *p* = 0.002), somatic complaints (54.9 ± 6.6 vs. 52.7 ± 4.5; *p* < 0.001), and DSM-oriented somatic problems (54.8 ± 7.1 vs. 52.4 ± 4.6; *p* < 0.001). Children with a diagnosis-ever of FA also showed significantly higher mean scores for internalizing problems (51.6 ± 9.7 vs. 49.5 ± 8.7; *p* = 0.033), anxious/depressed symptoms (54.8 ± 6.1 vs. 53.4 ± 5.0; *p* = 0.049), somatic complaints (54.7 ± 6.5 vs. 53.0 ± 4.9; *p* = 0.018), and DSM-oriented somatic problems (55.8 ± 7.4 vs. 52.7 ± 5.2; *p* = 0.002). Furthermore, children with current AD demonstrated significantly higher scores for internalizing problems (51.0 ± 9.1 vs. 49.5 ± 8.7; *p* = 0.043), somatic complaints (55.2 ± 5.9 vs. 52.9 ± 4.9; *p* < 0.001), and DSM-oriented somatic problems (55.5 ± 5.0 vs. 52.6 ± 5.0; *p* < 0.001) compared to those without. For current FA, children exhibited higher scores for somatic complaints (53.5 ± 6.1 vs. 53.1 ± 5.0; *p* = 0.041) and DSM-oriented somatic problems (56.2 ± 7.3 vs. 52.8 ± 5.3; *p* = 0.014) (Supplementary Table S4).

### 3.3. Allergic Disease Phenotypes

Children with a diagnosis-ever of AD only (without FA) were significantly associated with somatic complaints on the behavior problem scale (aOR, 2.09; 95% CI, 1.13–3.87) and DSM-oriented somatic problems (aOR, 2.35; 95% CI, 1.34–4.10). In contrast, children with a comorbid diagnosis-ever of both AD and FA showed significant associations with internalizing problems (aOR, 2.72; 95% CI, 1.28–5.75), somatic complaints (aOR, 4.33; 95% CI, 1.52–12.39), and externalizing problems (aOR, 2.27; 95% CI, 1.09–4.75) on the behavior problem scales. Furthermore, they demonstrated significantly higher risks for DSM-oriented anxiety problems (aOR, 2.43; 95% CI, 1.01–5.83), somatic problems (aOR, 8.90; 95% CI, 3.65–21.67), ADHD (aOR, 3.49; 95% CI, 1.13–10.83), and obsessive–compulsive disorder (OCD) on the special scale (aOR, 2.49; 95% CI, 1.05–5.88) (Table 2).

Neither current AD only (aOR, 1.80; 95% CI, 0.87–3.74) nor current FA only (aOR, 1.76; 95% CI, 0.38–8.19) was significantly associated with DSM-oriented somatic problems. Higher odds were observed among the small number of children with comorbid current AD and FA (*n* = 9; aOR, 37.35; 95% CI, 8.03–173.76) (Table 3).

### 3.4. Sex-Specific Differences

Sex-stratified analysis revealed distinct patterns in the associations between allergic disease phenotypes based on AD and FA status, and psychological or behavioral outcomes (Supplementary Tables S5–S8). Regarding lifetime diagnosis, male children with a diagnosis-ever of AD only (without FA) exhibited significant associations with somatic complaints on the behavior problem scale (aOR, 2.75; 95% CI, 1.23–6.14) and DSM-oriented somatic problems (aOR, 4.45; 95% CI, 1.81–10.94) (Supplementary Table S5). For male children with comorbid AD and FA, the associations were substantially more extensive, encompassing internalizing problems (aOR, 3.05; 95% CI, 1.21–7.68), somatic complaints (aOR, 8.86; 95% CI, 2.71–29.01), externalizing problems (aOR, 3.42; 95% CI, 1.40–8.38), rule-breaking behavior (aOR, 3.71; 95% CI, 1.23–11.21), and aggressive behavior (aOR, 3.33; 95% CI, 1.10–10.08). Furthermore, this comorbid group showed significantly higher risks for DSM-oriented somatic problems (aOR, 22.69; 95% CI, 7.35–76.32), ADHD (aOR, 4.59; 95% CI, 1.18–17.84), and conduct problems (aOR, 3.93; 95% CI, 1.15–13.42). In contrast, female children with a comorbid diagnosis-ever of AD and FA demonstrated a significant association only with the withdrawn subscale (aOR, 6.48; 95% CI, 1.22–34.50) (Supplementary Table S6).

Analysis of current disease status further highlighted these sex-specific disparities. In male children, current AD without current FA was associated with OCD (aOR, 0.26; 95% CI, 0.08–0.85), whereas comorbid current AD and FA were linked to rule-breaking behavior (aOR, 10.15; 95% CI, 1.60–64.36) and a remarkably elevated risk for DSM-oriented somatic problems (aOR, 66.82; 95% CI, 8.80–507.43) (Supplementary Table S7). While female children with comorbid current AD and FA also showed an association with DSM-oriented somatic problems (aOR, 14.11; 95% CI, 1.04–191.91) (Supplementary Table S8).

### 3.5. Combined Impact of Comorbid AD and FA on Somatic Problems

**Figure 1:**
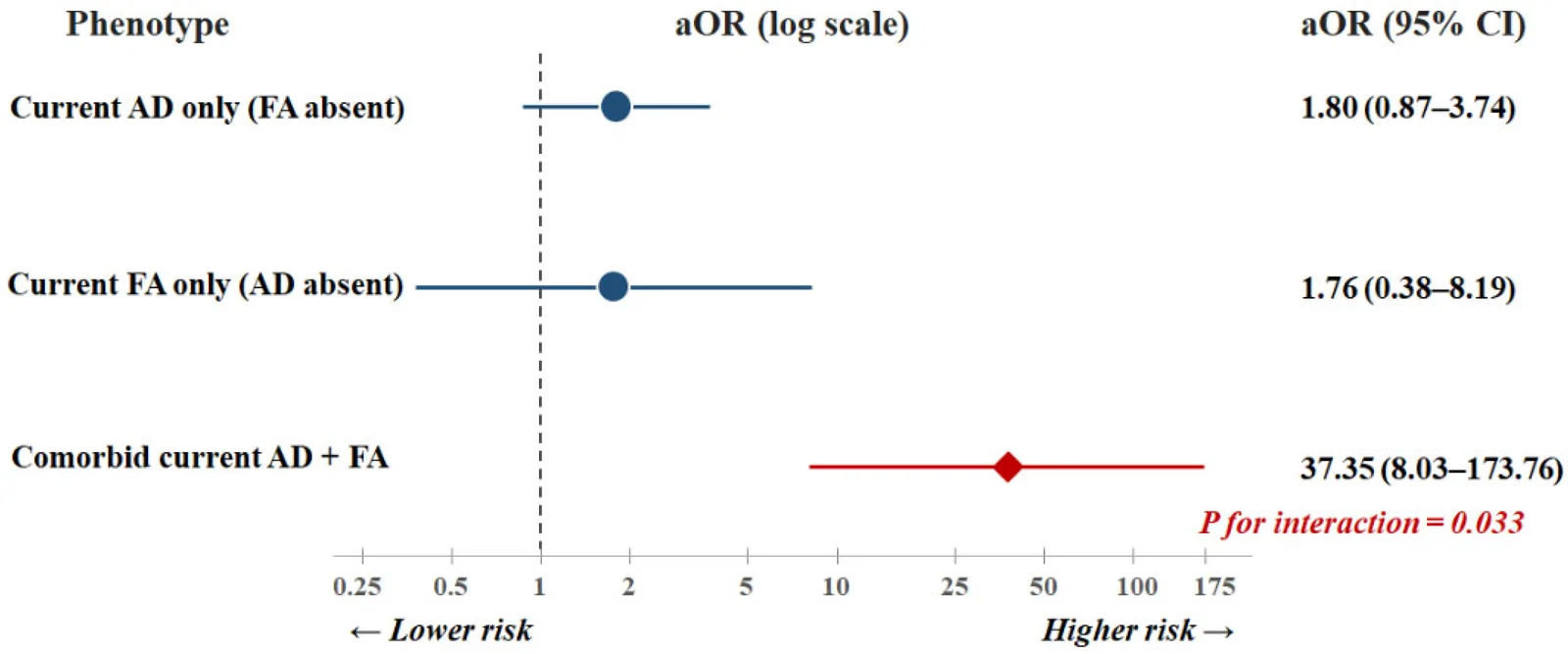
Interaction between current atopic dermatitis and food allergy on the risk of DSM-oriented somatic problems: a forest plot of adjusted odds ratios. Adjusted odds ratios (aOR) and 95% confidence intervals (CI) are shown on a log scale. The vertical dashed line indicates the null value (aOR = 1). The blue circles represent single-condition phenotypes (current AD only and current FA only), and the red diamond represents the comorbid current AD and FA group. Reference group: no current AD and no current FA. Models were adjusted for sex, gestational age, birth weight, parental allergy history, maternal education, household income, tobacco smoke exposure, pet ownership, and antibiotic use. *P* for interaction was derived from a multiplicative interaction term between current AD and current FA in logistic regression. AD, atopic dermatitis; FA, food allergy; DSM, Diagnostic and Statistical Manual of Mental Disorders.

The combined impact of AD and FA on DSM-oriented somatic problems was analyzed. An interaction was identified between current AD and current FA (*p* for interaction = 0.033), suggesting that the co-occurrence of both conditions may be associated with elevated risk of somatic symptoms beyond the individual contributions of either condition alone (Table 4, Figure 1).

### 3.6. Mediation and Interaction Effects of Respiratory Allergies

**Figure 2:**
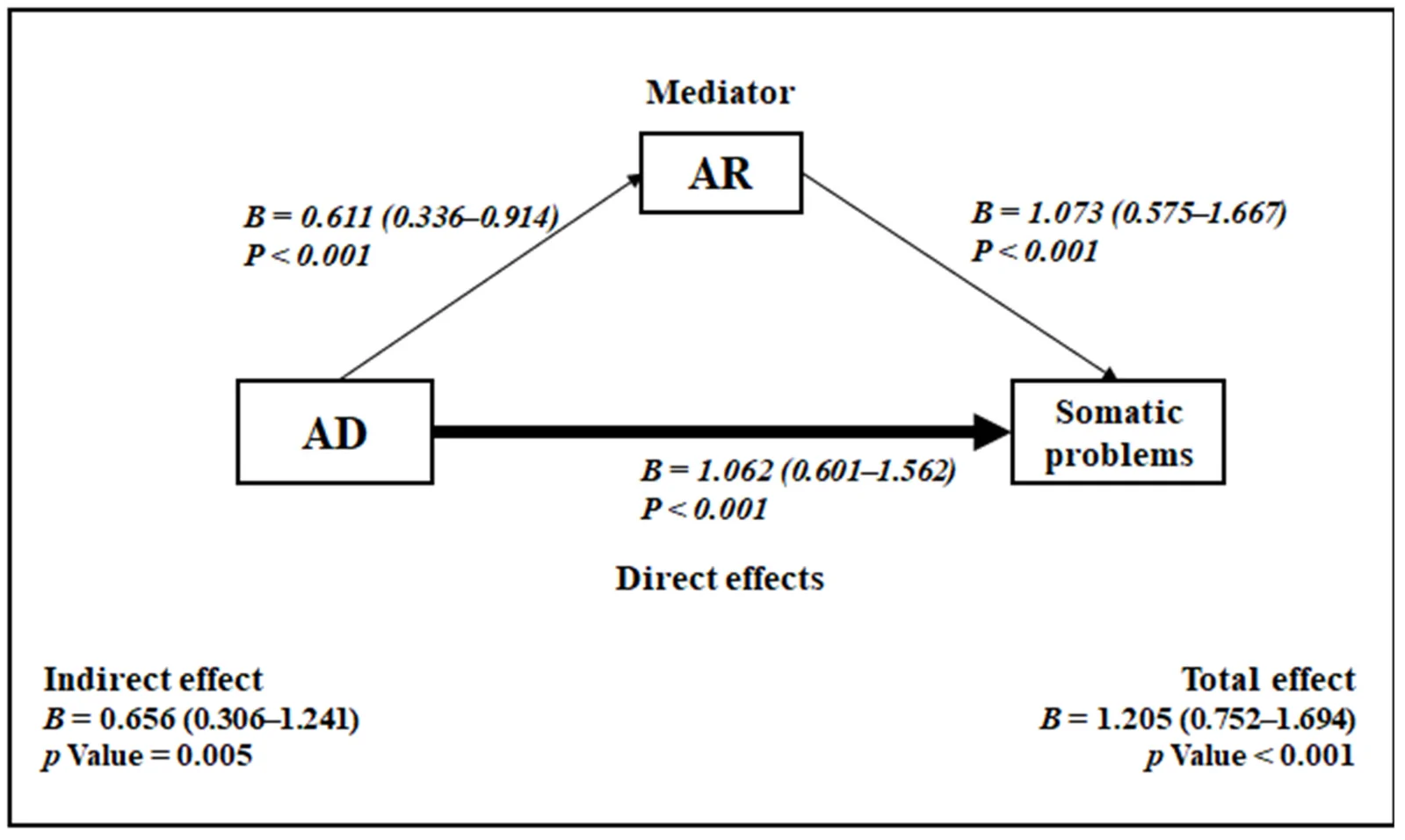
The mediating role of allergic rhinitis in the association between atopic dermatitis and somatic problems. AD and AR are binary variables. Numbers on arrows indicate *B* values with 95% confidence intervals. Abbreviations: *B*, unstandardised coefficient from logistic regression (log-odds scale); AD, atopic dermatitis; AR, allergic rhinitis.

Parallel mediation analyses were conducted using separate models for AD and FA to examine whether asthma and AR mediated their respective associations with somatic problems (Figure 2, Supplementary Tables S9 and S10). In the model evaluating lifetime history, the total effect of AD on somatic problems was significant (unstandardized coefficient [*B*] = 1.205; 95% CI: 0.752–1.694). Path analysis revealed that AD was significantly associated with asthma (a1: *B* = 0.612; 95% CI: 0.096–1.105) and AR (a2: *B* = 0.611; 95% CI: 0.336–0.914). Mediation tests demonstrated a significant indirect effect of AR (a2 × b2: *B* = 0.656; 95% CI: 0.306–1.241) (Figure 2), whereas the indirect effect of asthma did not reach statistical significance. Even after accounting for these mediators, the direct effect of AD remained robust (c: *B* = 1.062; 95% CI: 0.601–1.562). Similarly, for FA, a significant total effect on somatic problems was observed (*B* = 1.076; 95% CI: 0.381–1.770). Although FA was significantly linked to AR (a2: *B* = 0.554; 95% CI: 0.080–1.027), its indirect effect on AR was only marginally significant (a2 × b2: *B* = 0.584; 95% CI: −0.001–1.169) (Supplementary Tables S9 and S10).

In the current symptoms model, the total effects of both current AD (*B* = 1.078; 95% CI: 0.339–1.680) and current FA (*B* = 1.883; 95% CI: 0.977–2.788) on somatic problems were significant. Current AR symptoms significantly mediated the relationship with AD (a2 × b2: *B* = 0.503; 95% CI: 0.037–1.068) and showed a marginal mediating effect on FA (a2 × b2: *B =* 0.928; 95% CI, −0.054–1.909). In contrast, current asthma symptoms did not serve as a significant mediator in either model. Notably, the direct effects of the current AD and FA were the primary contributors to somatic outcomes (Tables S9 and S10). Furthermore, multivariate interaction analyses revealed no significant moderating effects of respiratory allergies on the associations among AD, FA, and somatic problems, respectively (Supplementary Tables S11 and S12).

### 3.7. Clinical Severity Assessment

The SCORAD index was compared across different disease phenotypes among children with AD aged 7 years. No statistically significant differences in SCORAD scores were observed based on the diagnosis of FA, current FA, or presence of somatic problems. Furthermore, no significant differences in the prevalence of moderate-to-severe AD were identified between the groups (Supplementary Table S13). These comparisons were based on the 37 children with an available SCORAD index; no inference regarding disease severity is drawn.

### 3.8. Biomarkers Across Allergic Disease Phenotypes

In the subsample of 633 children who attended a regional hospital, we analyzed serum CRP, total IgE, and blood eosinophil levels to identify biological variations across allergic disease phenotypes based on AD and FA status (Supplementary Table S14). In the analysis where allergic disease was defined as diagnosis-ever, serum CRP levels showed no significant variation across groups (*p* = 0.233). However, total IgE and eosinophil levels varied significantly according to the phenotype (*p* = 0.013 and *p* < 0.001, respectively). The highest levels were observed in children with comorbid AD and FA (total IgE: 5.52 ± 1.51 IU/mL; eosinophils: 6.01 ± 3.70%).

Similarly, when defining allergic disease as the current disease, CRP levels remained non-significant (*p* = 0.282), whereas total IgE and eosinophil levels varied significantly by phenotype (*p* < 0.001). Total IgE and eosinophil percentages peaked in children with comorbid AD and FA (5.85 ± 1.43 IU/mL and 7.30 ± 2.80%, respectively), whereas the lowest levels were recorded in children without both AD and FA.

## 4. Discussion

In this population-based birth cohort, AD and FA were each associated with somatic problems in 7-year-old children. Somatic problems were the outcome most consistently associated across allergic disease definitions. Among children with current comorbid AD and FA, a significant interaction was observed between the two conditions on somatic problems (*P* for interaction = 0.033), although the small size of this group (*n* = 9) renders the estimate imprecise and the finding exploratory. Additionally, AR statistically accounted for a portion of the association between AD and somatic problems. These results underscore the necessity for a holistic clinical approach that considers allergic multimorbidity in pediatric mental health.

Substantial epidemiological evidence has established that AD and FA have independent negative effects on pediatric mental health [25,26,27,28,29,30,31]. AD is well-recognized to elevate the risk of ADHD, anxiety, and depression through a combination of chronic pruritus, sleep fragmentation, and systemic Th2-mediated inflammation [25,26,27,28]. Similarly, FA imposes a unique psychological burden characterized by constant vigilance, fear of accidental exposure (anaphylaxis), and social isolation stemming from dietary restrictions [29,30,31]. However, despite the clinical reality of the atopic march—in which these conditions frequently coexist—most previous research has examined these diseases in isolation. Our study addresses this critical gap by demonstrating that the comorbidity of AD and FA is associated with a disproportionately greater psychological burden than either condition alone. These findings suggest that the cumulative burden of co-occurring AD and FA may reach a critical threshold, significantly elevating the risk of behavioral impairment in a manner that surpasses the individual impact of either condition.

Our analysis suggested some sex-related differences in the pattern of associations; however, the underlying mechanisms remain unclear. Traditionally, girls are more susceptible to internalizing symptoms, including somatic complaints, while boys are more frequently associated with externalizing problems and neurodevelopmental disorders such as ADHD [32,33,34]. In the present study, point estimates for the association between comorbid AD and FA and somatic problems were numerically higher in boys than in girls. However, the AD-by-FA interaction did not reach statistical significance within either sex stratum, and the relevant subgroups were very small; these observations are exploratory and are not interpreted further. Research on sex-specific psychological outcomes of AD or FA in prepubertal children remains scarce [35,36], and although sex hormones and sex-specific neuroimmune pathways have been proposed [37,38], such mechanisms are unlikely to operate before puberty and cannot be evaluated with the present data.

AD and FA are frequently associated with the subsequent development of respiratory comorbidities based on the concept of the atopic march [15,39,40]. While asthma and AR correlate strongly with various psychological disorders in children [7,41,42], whether they statistically account for such associations or contribute independently has been little examined. In the present data, AR statistically accounted for part of the association between AD and somatic problems. Since all variables in this study, including AD, FA, AR, asthma, and CBCL outcomes, were assessed concurrently at age 7, causal directionality cannot be established, and the mediation models should be read as statistical decompositions rather than as evidence of a causal pathway. AR symptoms such as nasal congestion and sleep fragmentation may act as a clinical bridge that intensifies psychological distress in children with AD [43,44]. Furthermore, the persistent direct association of AD is compatible with contributions from pruritus and systemic cytokines that are independent of respiratory allergic comorbidity, although the present data cannot test these mechanisms. Consequently, an integrated management strategy that addresses both the skin and the nasal system may be worth considering to mitigate the overall psychological burden.

Although AR statistically accounted for part of the association between AD and somatic problems, asthma did not show a significant indirect association. This discrepancy could be partly explained by the disparity in statistical power, as the number of children with asthma was significantly smaller than the number of children with AR. However, distinct clinical characteristics may play a critical role. Specifically, chronic upper airway inflammation and persistent nasal obstruction associated with AR often lead to near-daily sleep disruption [45,46]. Conversely, the symptoms of asthma tend to be more episodic and variable, resulting in a markedly different temporal pattern of somatic perception [47]. The persistent nature of AR symptoms, including nasal obstruction and its subsequent impact on sleep quality, may exert a more continuous and direct influence on a child’s somatic perception than the episodic manifestations of asthma. Chronic nasal symptoms are associated with somatic complaints such as headaches and fatigue [45,48,49]. These interpretations remain speculative and cannot be tested with the present data.

Our study had some limitations that warrant consideration. First, as this study was conducted in a general population-based cohort, the number of children with severe or comorbid allergic phenotypes was relatively small, particularly those with both current AD and FA (*n* = 9), resulting in wide confidence intervals that should be interpreted with caution. However, this is also a significant strength as it enables the generalization of our findings to a broader population. Second, the subgroup that underwent clinical assessments such as blood tests or SCORAD was smaller than the total cohort (biomarkers, *n* = 633; SCORAD, *n* = 37). Although baseline characteristics were mostly comparable except for a history of bronchiolitis, these analyses are exploratory, and no conclusions regarding disease severity or underlying mechanisms are drawn from them. Third, current FA was defined on the basis of parent-reported physician diagnosis together with maintenance of an elimination diet, rather than oral food challenge, which remains the diagnostic reference standard. Oral food challenge is not feasible at the scale of a nationwide birth cohort, but its absence is likely to have produced misclassification in both directions: children with outgrown or self-limited food allergy may have been classified as having current FA, whereas children with unrecognized or unreported allergy may have been classified as unaffected. The current FA definition, based on elimination diet maintenance for more than six months, may capture family management behaviour beyond simply reflecting active disease. Nevertheless, current food restriction serves as a clinically relevant proxy for the daily psychological burden experienced by families managing FA. This perceived burden may be a more direct driver of psychological distress than laboratory-confirmed diagnosis. Fourth, as allergic disease status and CBCL outcomes were both assessed at age 7, temporal directionality between allergic disease trajectories and psychological outcomes may not be fully established. However, given that AD and FA typically onset in infancy and early childhood while AR and asthma develop subsequently, and somatic problems become more clearly manifested during the school-age period, the findings of this study are both biologically and clinically plausible. Fifth, because both current FA status and K-CBCL scores were parent-reported, shared informant bias cannot be entirely excluded, as the same reporter’s perceptions may influence both exposure and outcome assessments. Nevertheless, parental reports are the most feasible and widely accepted method for assessing allergic disease status and psychological problems in young children at the population level, given that direct child self-report is not reliable at this age. Moreover, the domain-specific elevation observed in somatic problems rather than a global elevation across all K-CBCL domains argues against non-specific reporting bias as the sole explanation for our findings. Finally, this study conducted a large number of statistical tests across four allergic disease phenotype definitions and multiple CBCL subscales without formal correction for multiplicity, reflecting the exploratory nature of the research. Findings in secondary domains, including ADHD, anxiety, and OCD, should therefore be interpreted with caution and require replication in future confirmatory studies. Notably, when the Benjamini–Hochberg false discovery rate (FDR) correction was applied post hoc, the associations between allergic disease phenotypes and DSM-oriented somatic problems remained statistically significant across all exposure definitions (FDR-adjusted *p* = 0.017, 0.012, 0.017, and 0.008 for AD-ever, current FA, comorbid lifetime AD + FA, and comorbid current AD + FA, respectively), supporting the robustness of this finding. Somatic problems were not designated as the primary outcome in advance; the focus on this domain reflects the observed consistency of its associations, and the findings should therefore be regarded as hypothesis-generating. Despite these limitations, a major strength of this study is that it examines the combined psychological burden of allergic multimorbidity in a large-scale general population-based birth cohort, providing hypothesis-generating evidence that allergic multimorbidity may be accompanied by a greater burden of somatic problems than either condition alone.

## 5. Conclusions

Comorbid AD and FA were associated with higher odds of somatic problems than either condition alone in 7-year-old children, with a significant interaction observed between the two conditions; because only 9 children had concurrent AD and FA, this observation is exploratory and hypothesis-generating. AD was associated with somatic problems, with a portion of this association statistically accounted for by AR. Associations of asthma and AR with somatic problems were also examined; AR accounted for part of the association between AD and somatic problems. Because all exposures and outcomes were assessed concurrently, no causal inference is warranted. These results suggest that allergic multimorbidity—particularly coexisting AD and FA—may warrant integrated clinical attention that includes screening for somatic and psychological problems, alongside management of the skin and nasal symptoms, though prospective studies are needed to confirm these associations.

## Figures and Tables

**Table 1:** Association between individual allergic diseases and psychological/behavioral problems in children at 7 years of age.

	AD Diagnosis-Ever		FA Diagnosis-Ever		Current AD		Current FA	
	aOR * (95% CI)	*p* Value	aOR * (95% CI)	*p* Value	aOR * (95% CI)	*p* Value	aOR * (95% CI)	*p* Value
**Behavior problem scale**								
Total behavioral problems	1.05 (0.55–2.00)	0.889	0.54 (0.13–2.29)	0.405	0.69 (0.24–1.97)	0.491	-	
Internalizing Problems	1.39 (0.97–2.00)	0.072	1.59 (0.90–2.79)	0.111	0.95 (0.56–1.63)	0.853	1.51 (0.59–3.86)	0.387
Anxious/depressed	0.91 (0.56–1.48)	0.701	0.97 (0.43–2.20)	0.945	0.91 (0.46–1.82)	0.792	0.41 (0.06–3.08)	0.386
Withdrawn	1.03 (0.56–1.87)	0.937	1.65 (0.68–4.01)	0.266	1.25 (0.58–2.71)	0.574	1.49 (0.34–6.56)	0.596
Somatic complaints	**2.35 (1.33–4.13)**	**0.003**	1.85 (0.79–4.34)	0.160	1.48 (0.67–3.27)	0.336	2.27 (0.65–8.02)	0.201
Externalizing Problems	1.03 (0.72–1.46)	0.886	1.24 (0.71–2.17)	0.453	0.88 (0.53–1.47)	0.619	0.99 (0.36–2.69)	0.983
Rule-breaking behavior	1.24 (0.80–1.93)	0.340	0.86 (0.38–1.93)	0.706	1.24 (0.68–2.26)	0.490	1.15 (0.33–3.96)	0.825
Aggressive behavior	1.31 (0.81–2.12)	0.273	0.91 (0.38–2.18)	0.833	1.04 (0.52–2.10)	0.903	0.46 (0.06–3.45)	0.449
**DSM-oriented problem scale**								
Affective Problem	0.79 (0.43–1.46)	0.455	0.79 (0.28–2.25)	0.656	0.98 (0.43–2.22)	0.964	-	
Anxiety Problem	1.21 (0.77–1.90)	0.402	1.58 (0.80–3.14)	0.191	0.93 (0.48–1.80)	0.824	1.20 (0.35–4.14)	0.773
Somatic Problem	**2.94 (1.78–4.87)**	**<0.001**	**2.98 (1.45–6.14)**	**0.003**	**2.62 (1.39–4.93)**	**0.003**	**5.40 (2.09–13.97)**	**0.001**
ADHD	**1.95 (1.08–3.52)**	**0.027**	2.10 (0.90–4.92)	0.088	0.91 (0.35–2.35)	0.840	1.70 (0.39–7.47)	0.483
Oppositional Defiant Problem	0.93 (0.51–1.68)	0.799	0.63 (0.19–2.06)	0.442	0.82 (0.35–1.96)	0.656	-	
Conduct Problem	1.39 (0.85–2.30)	0.192	0.91 (0.36–2.35)	0.914	1.24 (0.62–2.49)	0.545	1.16 (0.26–5.06)	0.848
**Behavior problem special scale**								
OCD	1.05 (0.66–1.67)	0.848	1.38 (0.69–2.78)	0.364	0.45 (0.19–1.05)	0.066	1.25 (0.36–4.36)	0.724
PTSD	1.19 (0.65–2.16)	0.575	1.32 (0.50–3.46)	0.570	1.14 (0.50–2.59)	0.758	-	-
Cognitive problems	1.12 (0.55–2.27)	0.757	1.11 (0.33–3.73)	0.873	1.44 (0.59–3.53)	0.430	-	-

AD, atopic dermatitis; FA, food allergy; ADHD, attention-deficit hyperactivity disorder; DSM, Diagnostic and Statistical Manual of Mental Disorders. OCD, obsessive–compulsive disorder; PTSD, posttraumatic stress disorder. * Adjusted for sex, maternal education level, parental allergy history, history of second-hand smoking, residential area, economic status, and comorbid atopic diseases (current asthma and allergic rhinitis). Current AD was defined as both physician-diagnosed AD-ever and an eczema episode in the previous 12 months. Current FA was defined as both a physician-diagnosed food allergy and a diet eliminated during the previous 12 months. Current asthma was defined as both physician-diagnosed asthma and wheezing episodes during the previous 12 months in the medical records. Current AR was defined as both physician-diagnosed AR-ever and AR symptoms during the previous 12 months, based on medical records. Bold values indicate statistically significant associations (*p* < 0.05).

**Table 2:** Association between allergic disease phenotypes based on AD and FA status and psychological/behavioral problems in children at 7 years of age.

	AD Diagnosis-Ever (−), FA Diagnosis-Ever (−)		AD Diagnosis-Ever (−), FA Diagnosis-Ever (+)		AD Diagnosis-Ever (+), FA Diagnosis-Ever (−)		AD Diagnosis-Ever (+), FA Diagnosis-Ever (+)	
	aOR * (95% CI)	*p* Value	aOR * (95% CI)	*p* Value	aOR * (95% CI)	*p* Value	aOR * (95% CI)	*p* Value
**Behavior problem scale**								
Total behavioral problems	Reference		**-**	**-**	0.96 (0.48–1.91)	0.903	1.26 (0.29–5.57)	0.761
Internalizing Problems			1.02 (0.44–2.40)	0.960	1.23 (0.83–1.82)	0.298	**2.72 (1.28–5.75)**	**0.009**
Anxious/depressed			0.44 (0.10–1.90)	0.274	0.79 (0.46–1.35)	0.381	1.55 (0.58–4.76)	0.386
Withdrawn			1.47 (0.43–5.01)	0.542	0.87 (0.49–1.83)	0.866	1.80 (0.52–6.20)	0.352
Somatic complaints			1.06 (0.24–4.69)	0.944	**2.09 (1.13–3.87)**	**0.019**	**4.33 (1.52–12.39)**	**0.006**
Externalizing Problems			0.61 (0.25–1.48)	0.272	0.86 (0.58–1.26)	0.434	**2.27 (1.09–4.75)**	**0.029**
Rule-breaking behavior			0.42 (0.10–1.77)	0.235	1.15 (0.72–1.85)	0.564	1.55 (0.57–4.16)	0.389
Aggressive behavior			0.25 (0.03–1.87)	0.177	1.15 (0.68–1.93)	0.612	2.06 (0.75–5.61)	0.156
**DSM-oriented problem scale**								
Affective Problem			-	-	0.60 (0.30–1.21)	0.152	1.83 (0.61–5.46)	0.279
Anxiety Problem			0.96 (0.33–2.83)	0.946	1.05 (0.64–1.72)	0.853	**2.43 (1.01–5.83)**	**0.047**
Somatic Problem			1.11 (0.25–4.86)	0.892	**2.35 (1.34–4.10)**	**0.003**	**8.90 (3.65–21.67)**	**<0.001**
ADHD			1.77 (0.51–6.14)	0.371	1.83 (0.96–3.49)	0.067	**3.49 (1.13–10.83)**	**0.030**
Oppositional Defiant Problem			0.75 (0.17–3.26)	0.704	0.98 (0.53–1.81)	0.950	0.47 (0.06–3.53)	0.461
Conduct Problem			0.33 (0.04–2.42)	0.273	1.27 (0.74–2.16)	0.385	1.98 (0.67–5.90)	0.220
**Behavior problem special scale**								
OCD			0.57 (0.17–1.97)	0.377	0.82 (0.49–1.40)	0.471	**2.49 (1.05–5.88)**	**0.038**
PTSD			0.45 (0.06–3.39)	0.438	0.98 (0.50–1.91)	0.957	2.47 (0.81–7.52)	0.112
Cognitive problems			-		0.87 (0.39–1.92)	0.725	2.65 (0.75–9.42)	0.132

AD, atopic dermatitis; FA, food allergy; ADHD, attention-deficit hyperactivity disorder; DSM, Diagnostic and Statistical Manual of Mental Disorders. OCD, obsessive-compulsive disorder; PTSD, posttraumatic stress disorder. * Adjusted for sex, maternal education level, parental allergy history, history of second-hand smoking, residential area, economic status, and comorbid atopic diseases (current asthma and allergic rhinitis). Current AD was defined as both physician-diagnosed AD-ever and an eczema episode in the previous 12 months. Current FA was defined as both a physician-diagnosed food allergy and a diet eliminated during the previous 12 months. Current asthma was defined as both physician-diagnosed asthma and wheezing episodes during the previous 12 months in the medical records. Current AR was defined as both physician-diagnosed AR-ever and AR symptoms during the previous 12 months, based on medical records. Bold values indicate statistically significant associations (*p* < 0.05).

**Table 3:** Association between current allergic disease phenotypes based on AD and FA status and psychological/behavioral problems in children at 7 years of age.

	Current AD(−) Current FA(−)		Current AD(−) Current FA(+)		Current AD(+) Current FA(−)		Current AD(+) Current FA(+)	
	aOR * (95% CI)	*p* Value	aOR * (95% CI)	*p* Value	aOR * (95% CI)	*p* Value	aOR * (95% CI)	*p* Value
**Behavior problem scale**								
Total behavioral problems	Reference		**-**		0.77 (0.27–2.19)	0.621	**-**	
Internalizing Problems			1.81 (0.63–5.26)	0.273	0.97 (0.56–1.68)	0.911	0.88 (0.11–7.17)	0.903
Anxious/depressed			0.58 (0.08–4.44)	0.596	0.97 (0.49–1.94)	0.932	-	
Withdrawn			2.25 (0.49–10.32)	0.296	1.36 (0.63–2.97)	0.436	-	-
Somatic complaints			2.10 (0.45–9.72)	0.343	1.40 (0.60–3.25)	0.435	3.23 (0.37–28.43)	0.291
Externalizing Problems			0.71 (0.20–2.53)	0.601	0.82 (0.48–1.40)	0.465	1.88 (0.38–9.47)	0.442
Rule-breaking behavior			0.44 (0.06–3.43)	0.436	1.08 (0.57–2.06)	0.809	4.26 (0.84–21.74)	0.081
Aggressive behavior			0.62 (0.08–4.73)	0.641	1.10 (0.55–2.21)	0.795	-	
**DSM-oriented problem scale**								
Affective Problem			-		1.02 (0.45–2.32)	0.957	-	
Anxiety Problem			1.68 (0.46–6.05)	0.431	1.01 (0.51–1.95)	0.998	-	
Somatic Problem			1.76 (0.38–8.19)	0.472	1.80 (0.87–3.74)	0.116	**37.35 (8.03–173.76)**	**<0.001**
ADHD			1.06 (0.14–8.27)	0.954	0.76 (0.27–2.18)	0.610	3.49 (0.41–30.05)	0.255
Oppositional Defiant Problem			-		0.85 (0.35–2.02)	0.707	-	
Conduct Problem			0.72 (0.09–5.62)	0.757	1.16 (0.56–2.40)	0.698	2.90 (0.35–24.24)	0.325
**Behavior problem special scale**								
OCD			1.13 (0.25–5.25)	0.873	0.39 (0.16–1.00)	0.051	1.26 (0.15–10.82)	0.835
PTSD			-		1.19 (0.52–2.72)	0.682	-	-
Cognitive problems			-		1.49 (0.61–3.67)	0.384	-	-

AD, atopic dermatitis; FA, food allergy; ADHD, attention-deficit hyperactivity disorder; DSM, Diagnostic and Statistical Manual of Mental Disorders. OCD, obsessive–compulsive disorder; PTSD, posttraumatic stress disorder. * Adjusted for sex, maternal education level, parental allergy history, history of second-hand smoking, residential area, and economic status. Current AD was defined as both physician-diagnosed AD-ever and an eczema episode in the previous 12 months. Current FA was defined as both a physician-diagnosed food allergy ever and an elimination diet for >6 months at 7 years of age. Current asthma was defined as both physician-diagnosed asthma and wheezing episodes during the previous 12 months in the medical records. Current AR was defined as both physician-diagnosed AR-ever and AR symptoms during the previous 12 months, based on medical records. Bold values indicate statistically significant associations (*p* < 0.05).

**Table 4:** Interaction between atopic dermatitis and food allergy on the risk of somatic problems.

		aOR *^‡^ (95% CI)	*p* Value		aOR *^‡^ (95% CI)	*p* Value	*p* for Interaction
**Total participants**	**Stratified analyses**						
	AD diagnosis-ever (+) FA diagnosis-ever (−)	**2.35 (1.34–4.10)**	**0.003**	AD diagnosis-ever (+) FA diagnosis-ever (+)	**8.90 (3.65–21.67)**	**<0.001**	0.165
	AD diagnosis-ever (−) FA diagnosis-ever (+)	1.11 (0.25–4.86)	0.892				
	Current AD (+) Current FA (−)	1.80 (0.87–3.74)	0.116	Current AD (+) Current FA (+)	**37.35 (8.03–173.76)**	**<0.001**	**0.033**
	Current AD (−) Current FA (+)	1.76 (0.38–8.19)	0.472				
**Male**	AD diagnosis-ever (+) FA diagnosis-ever (−)	**4.45 (1.18–10.94)**	**0.001**	AD diagnosis-ever (+) FA diagnosis-ever (+)	**22.69 (7.35–76.32)**	**<0.001**	0.250
	AD diagnosis-ever (−) FA diagnosis-ever (+)	1.25 (0.14–10.89)	0.839				
	Current AD (+) Current FA (−)	1.81 (0.62–5.28)	0.279	Current AD (+) Current FA (+)	**66.82 (8.80–507.43)**	**<0.001**	0.100
	Current AD (−) Current FA (+)	2.49 (0.27–22.95)	0.422				
**Female**	AD diagnosis-ever (+) FA diagnosis-ever (−)	1.46 (0.66–3.24)	0.353	AD diagnosis-ever (+) FA diagnosis-ever (+)	2.58 (0.47–14.14)	0.274	0.858
	AD diagnosis-ever (−) FA diagnosis-ever (+)	1.37 (0.17–11.40)	0.769				
	Current AD (+) Current FA (−)	1.60 (0.56–4.62)	0.381	Current AD (+) Current FA (+)	**14.11 (1.04–191.91)**	**0.047**	0.367
	Current AD (−) Current FA (+)	1.73 (0.19–15.59)	0.625				

AD, atopic dermatitis; FA, food allergy. * Adjusted for sex, maternal education level, parental allergy history, history of second-hand smoking, residential area, economic status, and comorbid atopic diseases (current asthma and allergic rhinitis). ^‡^ Adjusted by maternal education levels, parental allergy history, history of second-hand smoking, residential area, economic status and comorbid atopic diseases (current asthma and current allergic rhinitis). Sex was excluded as a covariate from the sex-stratified model. Current AD was defined as both physician-diagnosed AD-ever and an eczema episode in the previous 12 months. Current FA was defined as both a physician-diagnosed food allergy and an elimination diet for >6 months at 7 years of age. Bold values indicate statistically significant associations (*p* < 0.05).

## Data Availability

The Panel Study on Korean Children (PSKC) datasets analyzed in this study are publicly available from the Korea Institute of Child Care and Education (KICCE) at https://panel.kicce.re.kr/pskc/module/rawDataManage/index.do (accessed on 24 June 2026) upon registration. The clinical and biomarker data obtained from the participating hospitals are not publicly available owing to privacy and ethical restrictions but are available from the corresponding author upon reasonable request.

## Supplementary Materials

The following supporting information can be downloaded at: https://www.mdpi.com/article/10.3390/children13081017/s1, Figure S1: Flow chart of the study. PSKC, Panel Study on Korean Children; AD, atopic dermatitis; FA, food allergy; SCORAD, SCORing Atopic Dermatitis; Table S1: Characteristics of the whole PSKC cohort stratified by attendance at a hospital for allergic testing; Table S2: Characteristics of participants with and without SCORAD index assessment among those with current atopic dermatitis; Table S3: Prevalence of allergic diseases in PSKC; Table S4: CBCL T-scores in children at 7 years of age according to food allergy; Table S5: Association between the allergic disease phenotypes based on AD and FA status and psychological/behavioral problems in male children at 7 years of age; Table S6: Association between the allergic disease phenotypes based on AD and FA status and psychological/behavioral problems in female children at 7 years of age; Table S7: Association between the current allergic disease phenotypes based on AD and FA status and psychological/behavioral problems in male children at 7 years of age; Table S8: Association between the current allergic disease phenotypes based on AD and FA status and psychological/behavioral problems in female children at 7 years of age; Table S9: Mediation analysis of asthma and allergic rhinitis in the relationship between atopic dermatitis and somatic problems; Table S10: Mediation analysis of asthma and allergic rhinitis in the relationship between food allergy and somatic problems; Table S11: Interactive effects of atopic dermatitis and respiratory allergies (asthma and allergic rhinitis) on somatic problems; Table S12: Interactive effects of food allergy and respiratory allergies (asthma and allergic rhinitis) on somatic problems; Table S13: SCORAD index in children with current AD at 7 years of age; Table S14: Serum C-reactive protein, total IgE, and eosinophil levels across allergy phenotypes.

## References

[1] Gutowska-Ślesik J., Samoliński B., Krzych-Fałta E. (2023). The increase in allergic conditions based on a review of literature. Postep. Dermatol. Alergol..

[2] Davis K.L., Claudio-Etienne E., Frischmeyer-Guerrerio P.A. (2024). Atopic dermatitis and food allergy: More than sensitization. Mucosal Immunol..

[3] Bawany F., Northcott C.A., Beck L.A., Pigeon W.R. (2021). Sleep Disturbances and Atopic Dermatitis: Relationships, Methods for Assessment, and Therapies. J. Allergy Clin. Immunol. Pract..

[4] Barbarot S., Silverberg J.I., Gadkari A., Simpson E.L., Weidinger S., Mina-Osorio P., Rossi A.B., Brignoli L., Mnif T., Guillemin I. (2022). The Family Impact of Atopic Dermatitis in the Pediatric Population: Results from an International Cross-sectional Study. J. Pediatr..

[5] Bilaver L.A., Chadha A.S., Doshi P., O’Dwyer L., Gupta R.S. (2019). Economic burden of food allergy: A systematic review. Ann. Allergy Asthma Immunol..

[6] Schmitt J., Buske-Kirschbaum A., Roessner V. (2010). Is atopic disease a risk factor for attention-deficit/hyperactivity disorder? A systematic review. Allergy.

[7] Lu Z., Chen L., Xu S., Bao Q., Ma Y., Guo L., Zhang S., Huang X., Cao C., Ruan L. (2018). Allergic disorders and risk of depression: A systematic review and meta-analysis of 51 large-scale studies. Ann. Allergy Asthma Immunol..

[8] Rodrigues J., Franco-Pego F., Sousa-Pinto B., Bousquet J., Raemdonck K., Vaz R. (2021). Anxiety and depression risk in patients with allergic rhinitis: A systematic review and meta-analysis. Rhinology.

[9] Marsland B.J., Trompette A., Gollwitzer E.S. (2015). The Gut-Lung Axis in Respiratory Disease. Ann. Am. Thorac. Soc..

[10] McKenzie C., Tan J., Macia L., Mackay C.R. (2017). The nutrition-gut microbiome-physiology axis and allergic diseases. Immunol. Rev..

[11] Xie Q.W., Dai X., Tang X., Chan C.H.Y., Chan C.L.W. (2019). Risk of Mental Disorders in Children and Adolescents With Atopic Dermatitis: A Systematic Review and Meta-Analysis. Front. Psychol..

[12] Xu X., Li S., Chen Y., Deng X., Li J., Xiong D., Xie H. (2025). Association between allergic diseases and mental health conditions: An umbrella review. J. Allergy Clin. Immunol..

[13] Casale T.B., Warren C., Gupta S., Schuldt R., Wang R., Iqbal A., Seetasith A., Gupta R. (2024). The mental health burden of food allergies: Insights from patients and their caregivers from the Food Allergy Research & Education (FARE) Patient Registry. World Allergy Organ. J..

[14] Bao Q., Chen L., Lu Z., Ma Y., Guo L., Zhang S., Huang X., Xu S., Ruan L. (2018). Association between eczema and risk of depression: A systematic review and meta-analysis of 188,495 participants. J. Affect. Disord..

[15] Lee S.Y., Kim S., Kang M.J., Song K.B., Choi E.J., Jung S., Yoon J.S., Suh D.I., Shin Y.H., Kim K.W. (2022). Phenotype of Atopic Dermatitis With Food Allergy Predicts Development of Childhood Asthma via Gut Wnt Signaling. Allergy Asthma Immunol. Res..

[16] Paller A.S., Spergel J.M., Mina-Osorio P., Irvine A.D. (2019). The atopic march and atopic multimorbidity: Many trajectories, many pathways. J. Allergy Clin. Immunol..

[17] Bantz S.K., Zhu Z., Zheng T. (2014). The Atopic March: Progression from Atopic Dermatitis to Allergic Rhinitis and Asthma. J. Clin. Cell. Immunol..

[18] Bahk J., Yun S.C., Kim Y.M., Khang Y.H. (2015). Impact of unintended pregnancy on maternal mental health: A causal analysis using follow up data of the Panel Study on Korean Children (PSKC). BMC Pregnancy Childbirth.

[19] Jung S., Suh D.I., Lee S.Y., Yoon J., Cho H.J., Kim Y.H., Yang S.I., Kwon J.W., Jang G.C., Sun Y.H. (2018). Prevalence, Risk Factors and Cutoff Values for Bronchial Hyperresponsiveness to Provocholine in 7-Year-Old Children. Allergy Asthma Immunol. Res..

[20] Jung S., Lee S.Y., Yoon J., Cho H.J., Kim Y.H., Suh D.I., Yang S.I., Kwon J.W., Jang G.C., Sun Y.H. (2020). Risk Factors and Comorbidities Associated With the Allergic Rhinitis Phenotype in Children According to the ARIA Classification. Allergy Asthma Immunol. Res..

[21] The International Study of Asthma and Allergies in Childhood (ISAAC) Steering Committee (1998). Worldwide variation in prevalence of symptoms of asthma, allergic rhinoconjunctivitis, and atopic eczema: ISAAC. Lancet.

[22] Achenbach T. (1991). Manual for the CBCL/4-18 and 1991 Profile.

[23] Oh K., Lee H., Hong K., Ha E. (1997). Korean Version of Child Behavior Checklist (K-CBCL).

[24] European Task Force on Atopic Dermatitis (1993). Severity scoring of atopic dermatitis: The SCORAD index. Consensus Report of the European Task Force on Atopic Dermatitis. Dermatology.

[25] Xu Y.C., Wang J.P., Zhu W.J., Li P. (2020). Childhood atopic dermatitis as a precursor for developing attention deficit/hyperactivity disorder. Int. J. Immunopathol. Pharmacol..

[26] Nygaard U., Riis J.L., Deleuran M., Vestergaard C. (2016). Attention-Deficit/Hyperactivity Disorder in Atopic Dermatitis: An Appraisal of the Current Literature. Pediatr. Allergy Immunol. Pulmonol..

[27] Wan J., Shin D.B., Syed M.N., Abuabara K., Lemeshow A.R., Gelfand J.M. (2023). Atopic dermatitis and risk of major neuropsychiatric disorders in children: A population-based cohort study. J. Eur. Acad. Dermatol. Venereol..

[28] Brandt E.B., Sivaprasad U. (2011). Th2 Cytokines and Atopic Dermatitis. J. Clin. Cell. Immunol..

[29] Kachru R. (2020). Psychosocial issues and quality of life associated with food allergy. J. Food Allergy.

[30] Bingemann T.A., LeBovidge J., Bartnikas L., Protudjer J.L.P., Herbert L.J. (2024). Psychosocial Impact of Food Allergy on Children and Adults and Practical Interventions. Curr. Allergy Asthma Rep..

[31] Teufel M., Biedermann T., Rapps N., Hausteiner C., Henningsen P., Enck P., Zipfel S. (2007). Psychological burden of food allergy. World J. Gastroenterol..

[32] Biondo F., Thunell C.N., Xu B., Chu C., Jia T., Ing A., Quinlan E.B., Tay N., Banaschewski T., Bokde A.L.W. (2022). Sex differences in neural correlates of common psychopathological symptoms in early adolescence. Psychol. Med..

[33] Álvarez-Voces M., Villar P., Almón-Pazos K., Romero E. (2025). Testing gender differences in psychological adjustment among early adolescents though a multigroup analysis of latent profiles. Sci. Rep..

[34] Loyer Carbonneau M., Demers M., Bigras M., Guay M.C. (2021). Meta-Analysis of Sex Differences in ADHD Symptoms and Associated Cognitive Deficits. J. Atten. Disord..

[35] Goldstein N.A., Aronin C., Kantrowitz B., Hershcopf R., Fishkin S., Lee H., Weaver D.E., Yip C., Liaw C., Saadia T.A. (2015). The prevalence of sleep-disordered breathing in children with asthma and its behavioral effects. Pediatr. Pulmonol..

[36] Ma Y., Tang J., Wen Y., Hu Y., Liang J., Jiang L., Xing Y., Song Y. (2022). Associations of sleep problems with asthma and allergic rhinitis among Chinese preschoolers. Sci. Rep..

[37] Gutiérrez-Brito J.A., Lomelí-Nieto J., Muñoz-Valle J.F., Oregon-Romero E., Corona-Angeles J.A., Hernández-Bello J. (2024). Sex hormones and allergies: Exploring the gender differences in immune responses. Front. Allergy.

[38] Cristallo M., Furci F., Casciaro M., Gangemi S., Nettis E. (2025). Sex Differences in Oxidative Stress Concerning Allergic Diseases. Biomolecules.

[39] Spergel J.M. (2010). From atopic dermatitis to asthma: The atopic march. Ann. Allergy Asthma Immunol..

[40] Lee E., Kim J.H., Lee S.Y., Lee S.H., Park Y.M., Oh H.Y., Yeom J., Ahn H.S., Yoo H.J., Kim B.S. (2025). Developmental trajectories of atopic dermatitis with multiomics approaches in the infant gut: COCOA birth cohort. J. Allergy Clin. Immunol..

[41] Edvinsson Sollander S., Fabian H., Sarkadi A., Salari R., Fält E., Dahlberg A., Feldman I., Durbeej N. (2021). Asthma and allergies correlate with mental health problems in preschool children. Acta Paediatr..

[42] Hou A., Silverberg J.I. (2021). Predictors and age-dependent pattern of psychologic problems in childhood atopic dermatitis. Pediatr. Dermatol..

[43] Sherrey J., Biggs S., Dorrian J., Martin J., Gold M., Kennedy D., Lushington K. (2022). Allergic disease, sleep problems, and psychological distress in children recruited from the general community. Ann. Allergy Asthma Immunol..

[44] Ramirez F.D., Chen S., Langan S.M., Prather A.A., McCulloch C.E., Kidd S.A., Cabana M.D., Chren M.M., Abuabara K. (2019). Association of Atopic Dermatitis With Sleep Quality in Children. JAMA Pediatr..

[45] Liu J., Zhang X., Zhao Y., Wang Y. (2020). The association between allergic rhinitis and sleep: A systematic review and meta-analysis of observational studies. PLoS ONE.

[46] D’Elia C., Gozal D., Bruni O., Goudouris E., Meira E.C.M. (2022). Allergic rhinitis and sleep disorders in children—Coexistence and reciprocal interactions. J. Pediatr..

[47] Venkatesan P. (2025). 2025 GINA report for asthma. Lancet Respir. Med..

[48] Everhart R.S., Kopel S.J., Esteban C.A., McQuaid E.L., Klein R., McCue C.E., Koinis-Mitchell D. (2014). Allergic rhinitis quality of life in urban children with asthma. Ann. Allergy Asthma Immunol..

[49] Gryglas A. (2016). Allergic Rhinitis and Chronic Daily Headaches: Is There a Link?. Curr. Neurol. Neurosci. Rep..

