# Supplementary material for: Associations of Comorbid Atopic Dermatitis, Food Allergy, and Respiratory Allergic Diseases with Somatic Problems in Children: A Birth Cohort Study
Source: Children (Basel). 2026 Jul 30;13(8):1017. doi: 10.3390/children13081017 (PMC13510927; doi:10.3390/children13081017)
Supplement: Supplementary file 1 [file children-13-01017-s001.zip › children-4422492-supplementary.pdf]

SUPPLEMENTARY ONLINE MATERIALS

Comorbid atopic dermatitis and food allergy are associated with increased somatic problem risk: a birth cohort study

Sungsu Jung[1], Jisun Yoon[2], Kyungmo Hong[3], Hea Young Oh[4], Eom Ji Choi[5], Min Jee Park[6], Song-I Yang[6], Eun Lee[7], Hyo-Bin Kim[8], So-Yeon Lee[9,10*], Soo-Jong Hong[4*], PSKC study group

Assessment of psychological and behavioral Problems

The CBCL is a widely utilized parent-reported instrument designed to assess emotional and behavioral symptoms in children [22]. The instrument comes in two versions according to the age range of the participants, and the version for aged 6–18 was used in this study. The checklist comprises several components: the Empirically Based Syndromes scale, Diagnostic and Statistical Manual of Mental Disorders (DSM)-oriented scales, and Social Problems scales. The Syndrome scale is further categorized into Internalizing Problems (anxious/depressed, somatic complaints, and withdrawn/depressed) and Externalizing Problems (rule-breaking and aggressive behaviors). Internalizing problems refer to overcontrolled, internally directed symptoms, whereas externalizing problems refer to undercontrolled, outwardly manifested behaviors. The DSM-oriented scales align with the diagnostic criteria for six domains: affective, anxiety, somatic, attention deficit/hyperactivity, oppositional defiance, and conduct problems. Specific scales measure obsessive-compulsive disorder (OCD), post-traumatic stress disorder (PTSD), and cognitive problems. The reliability and validity of the CBCL have been extensively documented in the Achenbach Manual [22], and the Korean version (K-CBCL) has demonstrated robust psychometric properties [23].

A key advantage of the CBCL is its use of standardized T-scores, which allow comparisons across different age groups and sexes. T-scores ranged from 23 to 100. For the Syndrome scales (total, internalizing, and externalizing), T-scores < 60 are considered non-clinical, 60–63 are borderline, and ≥ 64 are clinical. For the DSM-oriented and special scales, the cut-off points are ≤ 65 (non-clinical), 66–70 (borderline), and ≥ 71 (clinical). In the current analysis, psychological and behavioral problems were defined as scores falling within either the borderline or clinical ranges.

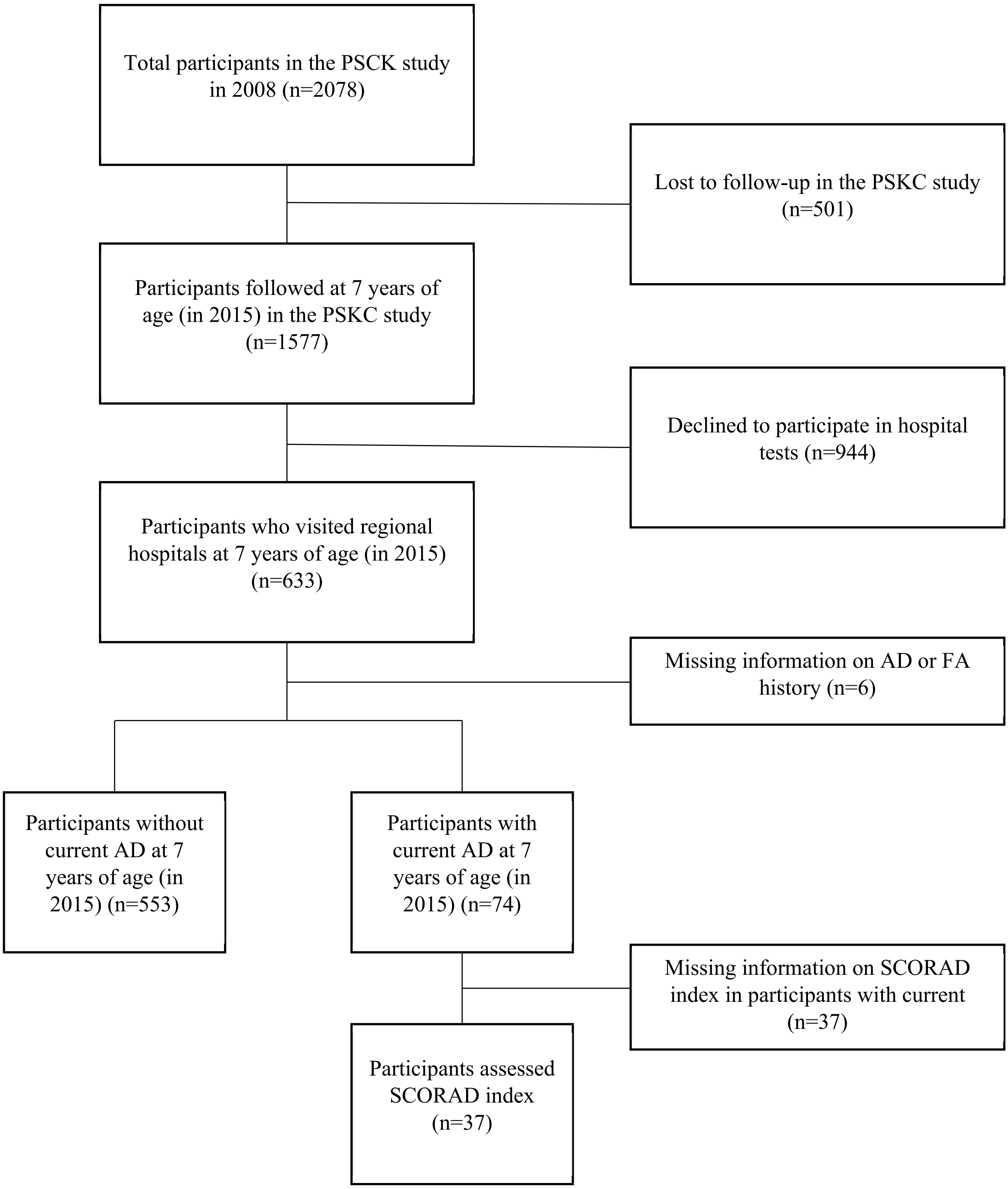

Figure S1. Flow chart of the study. PSKC, Panel Study on Korean Children; AD, atopic dermatitis; FA, food allergy; SCORAD, SCORing Atopic Dermatitis

Table S1. Characteristics of the whole PSKC cohort stratified by attendance at a hospital for allergic testing

Characteristics	Visiting participants		Non-visiting participants		*p* Value
	No./total (n=633)	No.(%)*	No./total (n=944)	No.(%)*	
Sex (Male)	342/633 (54.0)		467/944 (49.5)		0.076
Parental history of allergic disease	411/607 (67.7)		572/881 (64.9)		0.265
Environmental tobacco smoking	399/633 (63.0)		564/944 (59.7)		0.189
Maternal education level					0.446
≤ High school	185 (29.3)		290 (30.8)		
College	412 (65.2)		605 (64.1)		
Graduate school	35 (5.5)		46 (4.9)		
Past history of bronchiolitis	**154/632(24.4)**		**172/939(18.3)**		**0.004**
Frequent use of antibiotics in infancy (more than 3 days and 3 times)	124/633(19.6)		164/943(17.4)		0.268
Current pet ownership	48/627(7.7)		83/931(8.9)		0.380

*Data are presented as number (percentage) of patients unless otherwise indicated.

PSKC, Panel Study on Korean Children

Table S2. Characteristics of participants with and without SCORAD index assessment among those with current atopic dermatitis

Characteristics	With SCORAD index assessment	Without SCORAD index assessment	
	No./total No. (%)* (n=37)	No./total No. (%)* (n=37)	*p* Value
Sex (Male)	21/37 (56.8)	16/37 (43.2)	0.245
Parental history of allergic disease	32/36 (88.9)	26/34 (76.5)	0.168
Environmental tobacco smoking	24/37 (64.9)	24/37 (64.9)	1.000
Maternal education level			0.080
≤ High school	14/37 (37.8)	6/37 (16.2)	
college	7/37 (18.9)	16/37 (43.2)	
graduate school	16/37 (43.2)	15/37 (40.5)	
Past history of bronchiolitis	8/37 (21.6)	7/37 (18.9)	0.772
Frequent use of antibiotics in infancy (more than 3 days and 3 times)	7/37 (18.9)	7/37 (18.9)	1.000
Current pet ownership	1/36 (2.8)	3/36 (8.3)	0.614

*Data are presented as number (percentage) of patients unless otherwise indicated.

PSKC, Panel Study on Korean Children; SCORAD, SCORing atopic dermatitis

Table S3. Prevalence of allergic diseases in PSKC

		No./total No	(%)
Ever diagnosis	AD diagnosis ever	312/1563	20.0
	FA diagnosis ever	88/1573	5.6
	AD diagnosis ever (+), FA diagnosis ever (−)	274/1561	17.6
	AD diagnosis ever (−), FA diagnosis ever (+)	50/1561	3.2
	Both of AD and FA diagnosis, ever	38/1561	2.4
	Asthma diagnosis ever	91/1573	5.8
	AR diagnosis ever	708/1566	45.2
Current allergies at 7 years of age	Current AD	134/1563	8.6
	Current FA	31/1573	2.0
	Current AD (+), Current FA (−)	125/1561	8.0
	Current AD (−), Current FA (+)	22/1561	1.4
	Both of current AD and FA	9/1561	0.6
	Current asthma	24/1564	1.5
	Current AR	502/1558	32.2

PSKC, Panel Study on Korean Children; AD, atopic dermatitis; FA, food allergy; AR, allergic rhinitis

Current AD was defined as both physician-diagnosed AD-ever and an eczema episode in the previous 12 months.

Current FA was defined as both a physician-diagnosed food allergy and a diet eliminated during the previous 12 months.

Current asthma was defined as both physician-diagnosed asthma and wheezing episodes during the previous 12 months in the medical records.

Current AR was defined as both physician-diagnosed AR-ever and AR symptoms during the previous 12 months, based on medical records.

Table S4. CBCL T-scores in children at 7 years of age according to food allergy.

	AD diagnosis ever			FA diagnosis ever			Current AD			Current FA		
	No	Yes	*p* Value	No	Yes	*p* Value	No	Yes	*p* Value	No	Yes	*p* Value
**Behavior problem scale**												
Total behavioral problems	49.5±9.2	50.7±9.7	0.051	49.7±9.3	51.2±9.7	0.145	49.7±9.3	50.9±9.2	0.147	49.8±9.3	51.0±8.8	0.448
Internalizing Problems	49.3±8.6	51.0±9.3	**0.002**	49.5±8.7	51.6±9.7	**0.033**	49.5±8.7	51.0±9.1	0.043	49.6±8.7	51.0±9.8	0.387
Anxious/depressed	53.6±5.3	54.1±6.1	0.187	53.4±5.0	54.8±6.1	**0.049**	53.7±5.5	53.6±5.4	0.851	53.7±5.4	54.6±5.7	0.368
Withdrawn	53.4±4.9	53.5±5.5	0.770	53.4±5.0	54.0±4.9	0.263	53.4±5.0	53.2±5.4	0.556	53.4±5.0	53.9±4.7	0.569
Somatic complaints	52.7±4.5	54.9±6.6	**<0.001**	53.0±4.9	54.7±6.5	**0.018**	52.9±4.9	55.2±5.9	**<0.001**	53.1±5.0	53.5±6.1	**0.041**
Externalizing Problems	50.8±8.8	51.7±9.0	0.099	50.9±8.9	51.9±8.1	0.313	50.9±8.9	52.0±8.6	0.146	51.0±8.9	51.1±7.5	0.958
Rule-breaking behavior	54.8±5.2	55.1±5.8	0.442	54.8±5.3	55.3±5.3	0.413	54.8±5.3	55.1±5.6	0.644	54.8±5.3	55.3±5.2	0.613
Aggressive behavior	53.7±5.3	54.2±6.1	0.154	53.8±5.5	54.0±5.4	0.672	53.7±5.5	54.2±5.4	0.348	53.8±5.5	53.0±4.9	0.426
**DSM-oriented problem scale**												
Affective Problem	52.8±4.9	53.2±5.6	0.228	52.8±5.0	53.5±5.1	0.256	52.9±5.0	52.9±5.4	0.970	52.8±5.0	53.7±4.9	0.350
Anxiety Problem	53.9±5.8	54.5±6.7	0.204	53.9±5.9	55.4±7.3	0.076	54.0±6.0	54.0±6.1	0.979	54.0±6.0	55.7±7.5	0.234
Somatic Problem	52.4±4.6	54.8±7.1	**<0.001**	52.7±5.2	55.8±7.4	**0.002**	52.6±5.0	55.5±5.0	**<0.001**	52.8±5.3	56.2±7.3	**0.014**
ADHD	52.8±4.8	53.3±5.7	0.125	52.8±4.9	53.3±5.1	0.363	52.9±5.0	52.7±4.4	0.742	52.9±5.0	53.5±4.8	0.505
Oppositional Defiant Problem	53.6±5.3	53.7±5.6	0.787	53.6±5.4	53.6±4.9	0.251	53.6±5.4	53.6±5.0	0.925	53.7±5.4	52.4±4.0	0.188
Conduct Problem	53.0±5.0	53.3±6.2	0.430	53.1±5.3	53.3±5.4	0.649	53.1±5.2	53.3±6.0	0.591	53.1±5.3	53.1±5.1	0.997

**Behavior problem special scale**												
OCD	53.7±5.4	54.3±6.3	0.136	53.8±5.5	54.9±6.3	0.126	53.9±5.6	53.8±5.2	0.824	53.9±5.6	53.9±6.0	0.937
PTSD	53.3±5.0	53.7±6.0	0.312	53.3±5.1	54.0±5.6	0.244	53.4±5.1	53.3±5.6	0.768	53.3±5.2	54.3±5.1	0.297
Cognitive problems	52.0±4.7	52.4±5.8	0.292	52.1±4.9	52.5±5.0	0.443	52.1±4.8	52.4±5.6	0.422	52.1±4.9	52.4±4.6	0.751

AD, atopic dermatitis; FA, food allergy; AR, allergic rhinitis; ADHD, attention deficit hyperactivity disorder; DSM, Diagnostic and Statistical Manual of Mental Disorders.

OCD, obsessive-compulsive disorder; PTSD, posttraumatic stress disorder

*Adjusted for sex, maternal education level, parental allergy history, history of second-hand smoking, residential area, economic status, and comorbid atopic diseases (current asthma and allergic rhinitis).

Current AD was defined as both physician-diagnosed AD-ever and an eczema episode in the previous 12 months.

Current FA was defined as both a physician-diagnosed food allergy ever and an elimination diet for > 6 months at 7 years of age.

Current asthma was defined as both physician-diagnosed asthma and wheezing episodes during the previous 12 months in the medical records.

Current AR was defined as both physician-diagnosed AR-ever and AR symptoms during the previous 12 months, based on medical records.

Table S5. Association between the allergic disease phenotypes based on AD and FA status and psychological/behavioral problems in male children at 7 years of age

	AD diagnosis ever (−), FA diagnosis ever (−)		AD diagnosis ever (−), FA diagnosis ever (+)		AD diagnosis ever (+), FA diagnosis ever (−)		AD diagnosis ever (+), FA diagnosis ever (+)	
	aOR* (95% CI)	*p* Value	aOR* (95% CI)	*p* Value	aOR* (95% CI)	*p* Value	aOR* (95% CI)	*p* Value
**Behavior problem scale**								

Total behavioral problems		**-**	**-**	0.90 (0.31–2.56)	0.844	2.01 (0.40–10.15)	0.397
Internalizing Problems		1.28 (0.47–3.43)	0.630	1.17 (0.69–1.97)	0.503	**3.05 (1.21–7.68)**	**0.018**
Anxious/depressed		0.52 (0.12–2.32)	0.391	0.63 (0.31–1.27)	0.196	1.56 (0.50–4.90)	0.447
Withdrawn		2.31 (0.61–8.79)	0.219	0.71 (0.28–1.82)	0.474	0.87 (0.11–7.04)	0.900
Somatic complaints		0.70 (0.08–5.80)	0.740	**2.75 (1.23–6.14)**	**0.014**	**8.86 (2.71–29.01)**	**<0.001**
Externalizing Problems		0.58 (0.19–1.79)	0.351	0.85 (0.51–1.43)	0.547	**3.42 (1.40–8.38)**	**0.007**
Rule-breaking behavior		0.40 (0.05–3.14)	0.385	1.58 (0.82–3.06)	0.187	**3.71 (1.23–11.21)**	**0.020**
Aggressive behavior		0.37 (0.05–2.89)	0.342	1.01 (0.50–2.06)	0.977	**3.33 (1.10–10.08)**	**0.033**
**DSM-oriented problem scale**							
Affective Problem	Reference	-	-	0.65 (0.20–1.57)	0.382	2.03 (0.56–7.41)	0.283
Anxiety Problem		1.10 (0.31–3.98)	0.881	1.03 (0.52–2.04)	0.926	2.86 (0.98–8.37)	0.055
Somatic Problem		1.25 (0.14–10.86)	0.839	**4.45 (1.81–10.94)**	**0.001**	**22.69 (7.35–76.32)**	**<0.001**
ADHD		3.41 (0.87–13.33)	0.078	2.04 (0.81–5.11)	0.129	**4.59 (1.18–17.84)**	**0.028**
Oppositional Defiant Problem		0.67 (0.08–5.48)	0.711	0.61 (0.22–1.70)	0.341	-	-
Conduct Problem		-	-	1.10 (0.46–2.63)	0.830	**3.93 (1.15–13.42)**	**0.029**
**Behavior problem special scale**							
OCD		0.66 (0.19–2.32)	0.517	0.76 (0.42–1.38)	0.361	2.50 (0.97–6.46)	0.059
PTSD		0.65 (0.08–5.21)	0.683	1.02 (0.43–2.41)	0.971	2.46 (0.62–9.72)	0.201
Cognitive problems		-		1.49 (0.48–4.57)	0.490	2.12 (0.25–18.12)	0.494

AD, atopic dermatitis; FA, food allergy; AR, allergic rhinitis; ADHD, attention deficit hyperactivity disorder; DSM, Diagnostic and Statistical Manual of Mental Disorders.

OCD, obsessive-compulsive disorder; PTSD, posttraumatic stress disorder

*Adjusted for sex, maternal education level, parental allergy history, history of second-hand smoking, residential area, economic status, and comorbid atopic diseases (current asthma and allergic rhinitis).

Current AD was defined as both physician-diagnosed AD-ever and an eczema episode in the previous 12 months.

Current FA was defined as both a physician-diagnosed food allergy ever and an elimination diet for > 6 months at 7 years of age.

Current asthma was defined as both physician-diagnosed asthma and wheezing episodes during the previous 12 months in the medical records.

Current AR was defined as both physician-diagnosed AR-ever and AR symptoms during the previous 12 months, based on medical records.

Table S6. Association between the allergic disease phenotypes based on AD and FA status and psychological/behavioral problems in female children at 7 years of age

	AD diagnosis ever (−), FA diagnosis ever (−)	AD diagnosis ever (−), FA diagnosis ever (+)		AD diagnosis ever (+), FA diagnosis ever (−)		AD diagnosis ever (+), FA diagnosis ever (+)	
		aOR* (95% CI)	*p* Value	aOR* (95% CI)	*p* Value	aOR* (95% CI)	*p* Value
**Behavior problem scale**							
Total behavioral problems	Reference	-	-	1.02 (0.39–2.63.)	0.973	-	-
Internalizing Problems		0.69 (0.09–5.46)	0.729	1.26 (0.68–2.34)	0.470	2.64 (0.66–10.52)	0.170
Anxious/depressed		-	-	1.03 (0.43–2.46)	0.945	1.49 (0.18–12.57)	0.712
Withdrawn		-	-	1.20 (0.46–3.12)	0.703	**6.48 (1.22–34.50)**	**0.029**

Somatic complaints	2.70 (0.31–23.43)	0.367	1.30 (0.45–3.77)	0.629	-	-
Externalizing Problems	0.68 (0.15–3.15)	0.622	0.85 (0.46–1.58)	0.606	0.76 (0.16–3.68)	0.731
Rule-breaking behavior	0.44 (0.06–3.47)	0.434	0.82 (0.39–1.72)	0.597	-	-
Aggressive behavior	-	-	1.21 (0.54–2.69)	0.642	-	-
**DSM-oriented problem scale**						
Affective Problem	-	-	0.51 (0.15–1.78)	0.289	1.49 (0.17–12.79)	0.715
Anxiety Problem	0.88 (0.11–7.15)	0.903	1.05 (0.50–2.23)	0.893	1.89 (0.37–9.52)	0.442
Somatic Problem	1.37 (0.17–11.40)	0.769	1.46 (0.66–3.24)	0.353	2.58 (0.47–14.14)	0.274
ADHD	-	-	1.85 (0.69–4.96)	0.225	2.50 (0.26–24.07)	0.429
Oppositional Defiant Problem	0.81 (0.10–6.59)	0.846	1.13 (0.51–2.48)	0.764	0.98 (0.12–8.09)	0.984
Conduct Problem	0.67 (0.08–5.26)	0.699	1.35 (0.67–2.72)	0.407	-	-
**Behavior problem special scale**						
OCD	-	-	0.88 (0.28–2.80)	0.827	1.95 (0.20–19.14)	0.566
PTSD	-	-	0.86 (0.28–2.65)	0.799	2.65 (0.30–23.28)	0.380
Cognitive problems	-		0.45 (0.13–1.57)	0.209	2.90 (0.54–15.59)	0.214

AD, atopic dermatitis; FA, food allergy; AR, allergic rhinitis; ADHD, attention deficit hyperactivity disorder; DSM, Diagnostic and Statistical Manual of Mental Disorders. OCD, obsessive-compulsive disorder; PTSD, posttraumatic stress disorder

* Adjusted for maternal education level, parental allergy history, history of second-hand smoking, residential area, economic status, and comorbid atopic diseases (current asthma and allergic rhinitis). Sex was excluded as a covariate from the sex-stratified model.

Current AD was defined as both physician-diagnosed AD-ever and an eczema episode in the previous 12 months.

Current FA was defined as both a physician-diagnosed food allergy ever and an elimination diet for > 6 months at 7 years of age.

Current asthma was defined as both physician-diagnosed asthma and wheezing episodes during the previous 12 months in the medical records.

Current AR was defined as both physician-diagnosed AR-ever and AR symptoms during the previous 12 months, based on medical records.

Table S7. Association between the current allergic disease phenotypes based on AD and FA status and psychological/behavioral problems in male children at 7 years of age

	Current AD(−) Current FA(−)		Current AD(−) Current FA(+)		Current AD(+) Current FA(−)		Current AD(+) Current FA(+)	
	aOR* (95% CI)	*p* Value	aOR* (95% CI)	*p* Value	aOR* (95% CI)	*p* Value	aOR* (95% CI)	*p* Value
**Behavior problem scale**								
Total behavioral problems			-		1.08 (0.30–3.92)	0.906	-	
Internalizing Problems			2.31 (0.54–9.87)	0.259	0.95 (0.47–1.95)	0.892	1.64 (0.18–14.74)	0.660
Anxious/depressed			0.95 (0.11–7.92)	0.961	0.78 (0.32–1.92)	0.588	-	
Withdrawn			2.01 (0.23–17.96)	0.532	1.09 (0.35–3.34)	0.883	-	-
Somatic complaints			1.66 (0.19–14.85)	0.649	1.04 (0.34–3.23)	0.942	9.22 (0.86–98.28)	0.066
Externalizing Problems	Reference		0.90 (0.18–4.53)	0.900	0.85 (0.43–1.69)	0.647	1.17 (0.13–10.79)	0.888
Rule-breaking behavior			1.21 (0.14–10.26)	0.863	1.64 (0.73–3.67)	0.230	**10.15 (1.60–64.36)**	**0.014**
Aggressive behavior			1.27 (0.15–10.89)	0.827	1.13 (0.47–2.73)	0.779	-	
**DSM-oriented problem scale**								
Affective Problem			-		0.96 (0.32–2.88)	0.947	-	
Anxiety Problem			2.82 (0.54–14.56)	0.217	0.92 (0.37–2.28)	0.849	-	

Somatic Problem	2.49 (0.27–22.95)	0.422	1.81 (0.62–5.28)	0.279	**66.82 (8.80–507.43)**	**<0.001**
ADHD	2.64 (0.30–23.14)	0.380	0.66 (0.15–2.95)	0.582	5.28 (0.53–52.70)	0.157
Oppositional Defiant Problem	-		0.49 (0.11–2.24)	0.360	-	
Conduct Problem	3.32 (0.37–29.96)	0.285	1.52 (0.53–4.35)	0.433	4.48 (0.45–45.05)	0.203
**Behavior problem special scale**						
OCD	1.64 (0.32–8.30)	0.551	**0.26 (0.08–0.85)**	**0.026**	1.52 (0.17–14.06)	0.710
PTSD	-		1.17 (0.41–3.32)	0.773	-	-
Cognitive problems	-		2.82 (0.84–9.48)	0.093	-	-

AD, atopic dermatitis; FA, food allergy; AR, allergic rhinitis; ADHD, attention deficit hyperactivity disorder; DSM, Diagnostic and Statistical Manual of Mental Disorders.

OCD, obsessive-compulsive disorder; PTSD, posttraumatic stress disorder

* Adjusted for maternal education level, parental allergy history, history of second-hand smoking, residential area, economic status, and comorbid atopic diseases (current asthma and allergic rhinitis). Sex was excluded as a covariate from the sex-stratified model.

Current AD was defined as both physician-diagnosed AD-ever and an eczema episode in the previous 12 months.

Current FA was defined as both a physician-diagnosed food allergy ever and an elimination diet for > 6 months at 7 years of age.

Current asthma was defined as both physician-diagnosed asthma and wheezing episodes during the previous 12 months in the medical records.

Current AR was defined as both physician-diagnosed AR-ever and AR symptoms during the previous 12 months, based on medical records.

Table S8. Association between the current allergic disease phenotypes based on AD and FA status and psychological/behavioral problems in female children at 7 years of age

	Current AD(−) Current FA(−)	Current AD(−) Current FA(+)		Current AD(+) Current FA(−)		Current AD(+) Current FA(+)	
		aOR* (95% CI)	*p* Value	aOR* (95% CI)	*p* Value	aOR* (95% CI)	*p* Value
**Behavior problem scale**							
Total behavioral problems		**-**		0.31 (0.04–2.35)	0.254	**-**	
Internalizing Problems		2.29 (0.49–11.76)	0.319	0.91 (0.36–2.25)	0.831	-	-
Anxious/depressed		-	-	1.38 (0.46–4.14)	0.563	-	-
Withdrawn		4.99 (0.56–44.74)	0.151	1.61 (0.52–4.94)	0.406	-	-
Somatic complaints		4.23 (0.45–39.99)	0.209	1.76 (0.47–6.56)	0.398	-	
Externalizing Problems	Reference	0.46 (0.06–3.95)	0.482	0.75 (0.30–1.84)	0.527	1.95 (0.15–25.91)	0.613
Rule-breaking behavior		-	-	0.50 (0.15–1.71)	0.269	-	-
Aggressive behavior		-	-	0.81 (0.24–2.82)	0.744	-	
**DSM-oriented problem scale**							
Affective Problem		-		1.28 (0.36–4.60)	0.703	-	
Anxiety Problem		1.14 (0.13–11.69)	0.907	1.18 (0.44–3.20)	0.740	-	
Somatic Problem		1.73 (0.19–15.59)	0.625	1.60 (0.56–4.62)	0.381	**14.11 (1.04–191.91)**	**0.047**

ADHD	-	-	0.73 (0.15–3.49)	0.693	-	-
Oppositional Defiant Problem	-		0.99 (0.33–2.94)	0.987	-	
Conduct Problem	-	-	0.86 (0.29–2.55)	0.785	-	-
**Behavior problem special scale**						
OCD	-	-	0.89 (0.19–4.17)	0.885	-	-
PTSD	-		0.91 (0.20–4.05)	0.898	-	-
Cognitive problems	-		0.71 (0.15–3.23)	0.654	-	-

AD, atopic dermatitis; FA, food allergy; AR, allergic rhinitis; ADHD, attention deficit hyperactivity disorder; DSM, Diagnostic and Statistical Manual of Mental Disorders. OCD, obsessive-compulsive disorder; PTSD, posttraumatic stress disorder

* Adjusted for maternal education level, parental allergy history, history of second-hand smoking, residential area, economic status, and comorbid atopic diseases (current asthma and allergic rhinitis). Sex was excluded as a covariate from the sex-stratified model.

Current AD was defined as both physician-diagnosed AD-ever and an eczema episode in the previous 12 months.

Current FA was defined as both a physician-diagnosed food allergy ever and an elimination diet for > 6 months at 7 years of age.

Current asthma was defined as both physician-diagnosed asthma and wheezing episodes during the previous 12 months in the medical records.

Current AR was defined as both physician-diagnosed AR-ever and AR symptoms during the previous 12 months, based on medical records.

Table S9. Mediation analysis of asthma and allergic rhinitis in the relationship between atopic dermatitis and somatic problems

			Ever diagnosis		Current diseases	
	Pathway way	Description	*B* (95% CI)	*p* Value	*B* (95% CI)	*p* Value
Direct Effects	a1	AD → Asthma	0.612 (0.096, 1.105)	**0.012**	0.450 (−20.513, 1.543)	0.478
	a2	AD → AR	0.611 (0.336, 0.914)	**<0.001**	0.409 (0.024, 0.770)	**0.034**
	b1	Asthma → Somatic Problems	0.572 (−0.310, 1.300)	0.150	0.982 (−0.594, 2.100)	0.096
	b2	AR → Somatic Problems	1.073 (0.575, 1.667)	**<0.001**	1.230 (0.737, 1.769)	**<0.001**
	c	Direct Effect (AD → Somatic problems)	1.062 (0.601, 1.562)	**<0.001**	0.980 (0.251, 1.573)	**0.002**
Indirect Effects	a1×b1	Via Asthma	0.350 (−0.232, 0.972)	0.150	0.441 (−12.779, 2.430)	0.096
	a2×b2	Via AR	0.656 (0.306,1.241)	**0.005**	0.503 (0.037, 1.068)	**0.034**
Total Effect			1.205 (0.752, 1.694)	**<0.001**	1.078 (0.339, 1.680)	**<0.001**

B, unstandardized coefficient; CI, confidence interval; AD, atopic dermatitis; FA, food allergy; AR, allergic rhinitis.

Table S10. Mediation analysis of asthma and allergic rhinitis in the relationship between food allergy and somatic problems

			Ever diagnosis		Current diseases	
	Pathway way	Description	*B* (95% CI)	*p* Value	*B* (95% CI)	*p* Value
Direct Effects	a1	FA → Asthma	0.374 (−0.406, 1.154)	0.347	1.686 (0.162, 3.210)	**0.030**
	a2	FA → AR	0.554 (0.080, 1.027)	**0.022**	0.814 (0.035, 1.593)	**0.041**
	b1	Asthma → Somatic Problems	0.637 (−0.135, 1.408)	0.106	0.848 (−0.348, 2.044)	0.165
	b2	AR → Somatic Problems	1.055 (0.506, 1.604)	**<0.001**	1.139 (0.626, 1.653)	**<0.001**
	c	Direct Effect (FA → Somatic problems)	0.971 (0.267, 1.675)	**0.007**	1.671 (0.733, 2.609)	**<0.001**
Indirect Effects	a1×b1	Via Asthma	0.238 (−0.336,0.812)	0.416	1.430 (−0.965, 3.825)	0.242
	a2×b2	Via AR	0.584 (−0.001, 1.169)	0.050	0.928 (−0.054, 1.909)	0.064
Total Effect			1.076 (0.381, 1.770)	**0.002**	1.883 (0.977, 2.788)	**<0.001**

B, unstandardized coefficient; CI, confidence interval; AD, atopic dermatitis; FA, food allergy; AR, allergic rhinitis.

Table S11. Interactive effects of atopic dermatitis and respiratory allergies (asthma and allergic rhinitis) on somatic problems

		aOR* (95% CI)	*p* Value		aOR* (95% CI)	*p* Value	*p* for interaction
	**Stratified analyses**						
Asthma	AD diagnosis ever (+)<br>Asthma diagnosis ever (−)	**3.46 (2.04–5.86)**	**<0.001**	AD diagnosis ever (+)<br>Asthma diagnosis ever (+)	**5.40 (1.69–17.33)**	**<0.001**	0.361
	AD diagnosis ever (−)<br>Asthma diagnosis ever (+)	**3.21 (1.18–8.76)**	**0.023**				
	Current AD (+)<br>Current asthma (−)	**2.87 (1.52–5.43)**	**0.001**	Current AD (+)<br>Current asthma (+)	**30.29 (2.18–421.53)**	**0.011**	0.615
	Current AD (−)<br>Current asthma (+)	**4.90 (1.28–18.67)**	**0.020**				
AR	AD diagnosis ever (+)<br>AR diagnosis ever (−)	**2.60 (0.97–7.01)**	**0.059**	AD diagnosis ever (+)<br>AR diagnosis ever (+)	**7.68 (3.79–15.54)**	**<0.001**	0.781
	AD diagnosis ever (−)<br>AR diagnosis ever (+)	**2.51 (1.27–4.93)**	**0.008**				
	Current AD (+)	**4.26 (1.62–11.19)**	**0.003**	Current AD (+)	**7.36 (3.09–17.50)**	**<0.001**	0.239

Current AR (−)			Current AR (+)
Current AD (−)<br>Current AR (+)	**3.70 (2.08–6.56)**	**<0.001**	

AD, atopic dermatitis; AR, allergic rhinitis; FA, food allergy

* Adjusted for sex, maternal education level, parental allergy history, history of secondhand smoke, residential area, and economic status. Comorbid atopic diseases (current asthma and current allergic rhinitis) were excluded as a covariate in the interactive effects models.

Table S12. Interactive effects of food allergy and respiratory allergies (asthma and allergic rhinitis) on somatic problems

		aOR* (95% CI)	*p* Value		aOR* (95% CI)	*p* Value	*p* for interaction
	**Stratified analyses**						
**Asthma**	FA diagnosis ever (+)<br>Asthma diagnosis ever (−)	**2.48 (1.12–5.54)**	**0.026**	FA diagnosis ever (+)<br>Asthma diagnosis ever (+)	**15.85 (3.32–75.65)**	**<0.001**	0.238
	FA diagnosis ever (−)<br>Asthma diagnosis ever (+)	2.02 (0.82–4.96)	0.127				
	Current FA (+)<br>Current asthma (−)	**7.49 (2.93–19.15)**	**<0.001**	Current FA (+)<br>Current asthma (+)	-	-	-
	Current FA (−)<br>Current asthma (+)	**7.04 (2.11–23.41)**	**0.001**				
**AR**	FA diagnosis ever (+)<br>AR diagnosis ever (−)	-	-	FA diagnosis ever (+)<br>Asthma diagnosis ever (+)	**9.70 (4.13–22.79)**	**<0.001**	0.998
	FA diagnosis ever (−)<br>AR diagnosis ever (+)	**2.51 (1.43–4.41)**	**0.001**				

Current FA (+)<br>Current AR (−)	**6.62 (1.32–33.14)**	**0.021**	Current FA (+)<br>Current asthma (+)	**14.63 (4.53–47.21)**	**<0.001**	0.686
Current FA (−)<br>Current AR (+)	**3.33 (1.96–5.66)**	**<0.001**				

AD, atopic dermatitis; AR, allergic rhinitis; FA, food allergy

* Adjusted for sex, maternal education level, parental allergy history, history of secondhand smoke, residential area, and economic status. Comorbid atopic diseases (current asthma and current allergic rhinitis) were excluded as a covariate in the interactive effects models.

Table S13. SCORAD index in children with current AD at 7 years of age

	No/total No (%)	Number of participants evaluated SCORAD index	SCORAD index		Moderate to severe AD (SCORAD index ≥ 25)	
			Median (interquartile range)	*p* Value	No/total No (%)	*p* Value
Current AD with FA diagnosis ever	45/1556 (2.9)	15	19.2 (0.0–29.0)	0.680	4/15 (8.9)	1.000
Current AD without FA diagnosis ever	89/1556 (5.7)	22	18.1 (2.8–37.5)		7/22 (7.9)	
Current AD with current FA	9/1561 (0.4)	3	19.2 (0.0–19.2)	1.000	1/3 (33.3)	1.000
Current AD without current FA	125/1561 (8.0)	34	18.9 (0.0–31.0)		10/34 (29.4)	
Current AD with somatic problems	15/1555 (1.0)	6	11.5 (0.0–49.5)	0.794	2/6 (33.3)	1.000
Current AD without somatic problems	119/1555 (7.7)	31	19.2 (0.0–29.0)		9/31 (29.0)	

AD, atopic dermatitis; FA, food allergy; SCORAD, SCORing atopic dermatitis

Mann–Whitney *U* tests were used for comparisons of continuous variables between groups.

Chi-square tests (or Fisher's exact tests) were used to compare categorical variables between groups.

Current AD was defined as both physician-diagnosed AD-ever and an eczema episode in the previous 12 months.

Current FA was defined as both a physician-diagnosed food allergy ever and an elimination diet for > 6 months at 7 years of age.

Table S14. Serum C-reactive protein, total IgE, and eosinophil levels across allergy phenotypes

	Diagnosis ever					Current diseases				
	**AD (−), FA (−) (N=444)**	**AD (−), FA (+) (N=21)**	**AD (+), FA (−) (N=142**	**AD (+), FA (+) (N=19)**	***p* Value (diagnosis ever)**	**AD (−) FA (−) (N=541)**	**AD (−) FA (+) (N=11)**	**AD (+) FA (−) (N=69)**	**AD (+) FA (+) (N=5)**	***p* Value (Current diseases)**
CRP (mg/dL)	1.10±2.17	0.56±0.38	1.09±2.33	2.10±5.04	0.233	1.07±2.07	0.59±0.53	1.64±3.95	0.52±0.30	0.282
In$^{Total\ IgE}$ (IU/mL)	4.60±1.35†‡	4.67±1.32	4.91±1.54†	5.52±1.51‡	0.013	4.58±1.39†‡	5.38±1.13	5.46±1.30†	5.85±1.43‡	<0.001
Eosinophil (%)	3.63±2.81†‡	4.45±3.63	4.95±4.25†	6.01±3.70‡	<0.001	3.78±3.02†‡	5.25±4.08	5.52±4.63†	7.30±2.80‡	<0.001

AD, atopic dermatitis; FA, food allergy; CRP, C-reactive protein.

*AD (−) and FA (−) vs AD (−) and FA (+)

†AD (−) and FA (−) vs AD (+) and FA (−)

‡AD (−) and FA (−) vs AD (+) and FA (+)

§AD (−) and FA (+) vs AD (+) and FA (−)

‖AD (−) and FA (+) vs AD (+) and FA (+)

¶AD (+) and FA (−) vs AD (+) and FA (+)
